# “Inverse” thermoresponse: heat-induced double-helix formation of an ethynylhelicene oligomer with tri(ethylene glycol) termini†
†Electronic supplementary information (ESI) available. See DOI: 10.1039/c5sc04959h


**DOI:** 10.1039/c5sc04959h

**Published:** 2016-02-12

**Authors:** Nozomi Saito, Higashi Kobayashi, Masahiko Yamaguchi

**Affiliations:** a Department of Organic Chemistry , Graduate School of Pharmaceutical Sciences , Tohoku University , Sendai , Japan . Email: yama@m.tohoku.ac.jp; b Tohoku University Frontier Research Institute for Interdisciplinary Science , Sendai , Japan

## Abstract

An ethynylhelicene oligomer [(*M*)-d-**4**]-C_12_-TEG with six tri(ethylene glycol) (TEG) groups at the termini was synthesized, and double-helix formation was studied using CD, UV-Vis, vapor pressure osmometry, dynamic light scattering, and ^1^H NMR. [(*M*)-d-**4**]-C_12_-TEG reversibly changed its structure between a double helix and a random coil in response to heating and cooling in aromatic solvents, non-aromatic polar organic solvents, and aqueous solvent mixtures of acetone/water/triethylamine. Notably, [(*M*)-d-**4**]-C_12_-TEG in acetone/water/triethylamine (1/2/1) formed a double helix upon heating and disaggregated into random coils upon cooling. The double helix/random coil ratio sharply changed in response to temperature changes. This is an unprecedented “inverse” thermoresponse, which is opposite to the “ordinary” thermoresponse in molecular dimeric aggregate formation. This phenomenon was explained by the dehydration of the terminal TEG groups and the formation of condensed triethylamine domains upon heating.

## Abstract 2

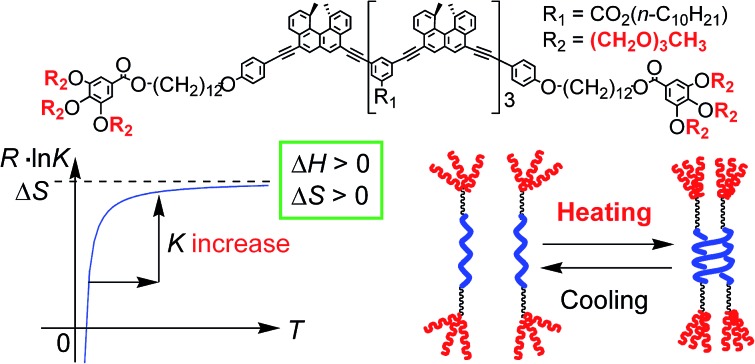
Ethynylhelicene oligomers with TEG terminal groups showed a unique thermoresponse in aqueous solvents: double-helix formation upon heating and disaggregation upon cooling.

## Introduction

**Fig. 1:**
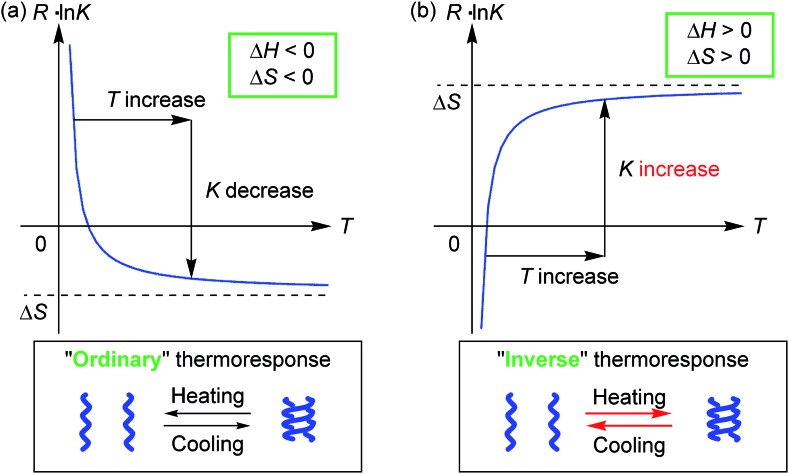
Graphical representation of the relationship between thermodynamic parameters and the (a) “ordinary” or (b) “inverse” thermoresponse.

Molecules form dimeric aggregates in solution upon cooling and disaggregate upon heating,^1^–^5^ and such a thermoresponse is termed an “ordinary” thermoresponse in this study. DNA is a typical example of a biological molecule that exhibits the “ordinary” thermoresponse and forms double helices upon cooling and random coils upon heating.^6^ A dimeric molecular aggregation **A** + **A** → **A_2_** is generally an exothermic process with a negative enthalpy change Δ*H* < 0, because **A_2_** is a structure with less internal energy or enthalpy than 2**A**. Entropy also decreases in dimeric aggregation, which shows a negative entropy change Δ*S* < 0, because the freedom in molecular motion is decreased in **A_2_** compared with 2**A**. The Gibbs free energy Δ*G* = Δ*H* – *T*Δ*S* increases with an increase in temperature *T*, because of Δ*H* > 0. Consequently, the concentration of **A_2_** decreases upon heating, and the equilibrium moves toward dissociation to give 2**A** according to the equations Δ*G* = –*RT* ln *K* and *R* ln *K* = –Δ*H*/*T* + Δ*S* (Fig. 1a), where *K* and *R* are the equilibrium constant and gas constant, respectively. The “ordinary” thermoresponse is widely observed for molecular dimeric aggregates.

In contrast, molecules that exhibit the “inverse” thermoresponse in dimeric aggregation are also conceivable, for which heating induces aggregation and cooling induces disaggregation. Hypothetically, this phenomenon can occur in an endothermic process with a positive enthalpy change Δ*H* > 0, and the process is accompanied by an increase in freedom in molecular mobility with a positive entropy change Δ*S* > 0. Consequently, the equilibrium moves toward aggregation to form **A_2_** upon heating according to the equations Δ*G* = –*RT* ln *K* and *R* ln *K* = –Δ*H*/*T* + Δ*S* (Fig. 1b), because of –Δ*H*/*T* < 0. However, such a thermoresponse is counter-intuitive. The dimeric aggregation of synthetic molecules exhibiting the “inverse” thermoresponse, being a molecular-level phenomenon, has essentially not been observed.^7^

**Scheme 1:**
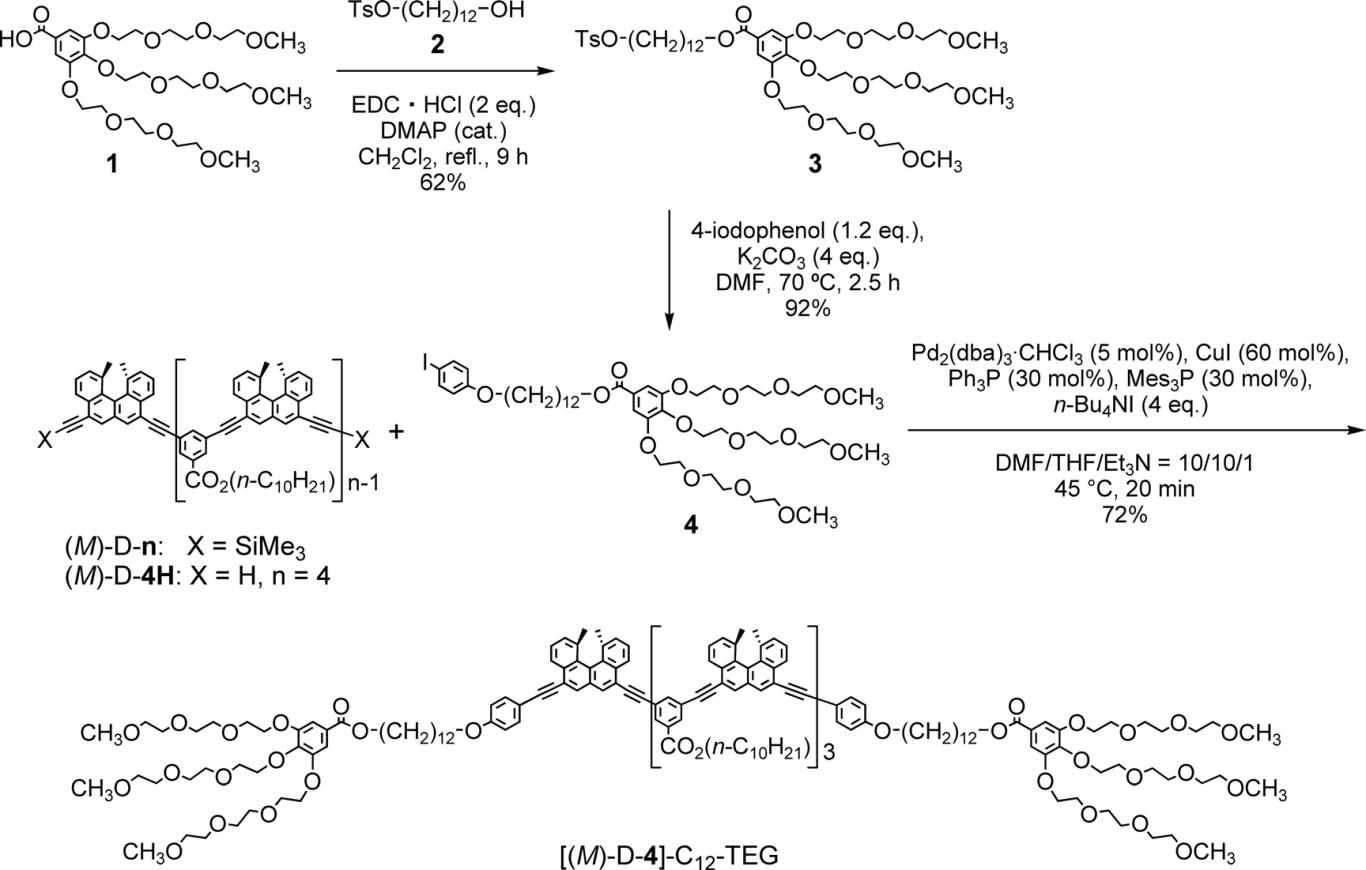
Synthesis of [(*M*)-d-**4**]-C_12_-TEG.

The “ordinary” and “inverse” thermoresponses are complementary, and the development of the latter will largely broaden the use of thermoresponsive materials. Here, we report the synthesis of [(*M*)-d-**4**]-C_12_-TEG, which is an ethynylhelicene tetramer with tri(ethylene glycol) (TEG) groups at its termini (Scheme 1), and it has a notable “inverse” thermoresponse: [(*M*)-d-**4**]-C_12_-TEG aggregated to form a double helix upon heating and disaggregated to give a random coil upon cooling in an aqueous solvent mixture of acetone/water/triethylamine. A sharp transition between the double helix and the random coil occurred due to temperature changes.

## Results and discussion

Our previous studies showed that (*M*)-d-**n**(ref. 5) exhibited the “ordinary” thermoresponse. [(*M*)-d-**4**]-C_12_-TEG, with six TEG groups at both termini, was designed in this study to examine aggregation behavior in aqueous solvents. Compound **4** with a TEG moiety was obtained from benzoic acid **1** (ref. 8) in 2 steps, which was then connected to the termini of the ethynylhelicene tetramer (*M*)-d-**4H**5*e* by Sonogashira coupling (Scheme 1).

[(*M*)-d-**4**]-C_12_-TEG showed the “ordinary” thermoresponse in organic solvents; it formed double helices upon cooling and disaggregated into random coils upon heating. Solutions of [(*M*)-d-**4**]-C_12_-TEG were heated at 60 °C or 40 °C for disaggregation then cooled, and their circular dichroism (CD) and UV-Vis spectra were obtained. The CD spectra in chloroform (5 × 10^–4^ M) at 40, 25, and 5 °C showed weak Cotton effects mirror-imaged to the typical random-coil state of (*P*)-ethynylhelicene tetramers5*a* (ESI Fig. S1a†). On the other hand, [(*M*)-d-**4**]-C_12_-TEG showed an intense CD as well as a hypochromic shift in the UV-Vis spectrum in trifluoromethylbenzene (1 × 10^–3^ M) upon cooling to 5 and –10 °C (ESI Fig. S1b†). The apparent molecular weight of the double helix obtained by vapor pressure osmometry (VPO) studies (trifluoromethylbenzene, 40 °C) above 1 × 10^–3^ M was twice as large as the calculated molecular weight of [(*M*)-d-**4**]-C_12_-TEG (3726.7) (ESI Fig. S2 and Table S2†). The results indicated the formation of double helices of [(*M*)-d-**4**]-C_12_-TEG in trifluoromethylbenzene. The reversible structural change between random coils and double helices was examined for Δ*ε* at 360 nm (trifluoromethylbenzene, 1 × 10^–3^ M) by repeating the cycle of heating to 60 °C and cooling to 5 °C (ESI Fig. S1c†). Δ*ε*_360_ increased upon heating and decreased upon cooling: the “ordinary” thermoresponse was observed in the aromatic solvent.

**Fig. 2:**
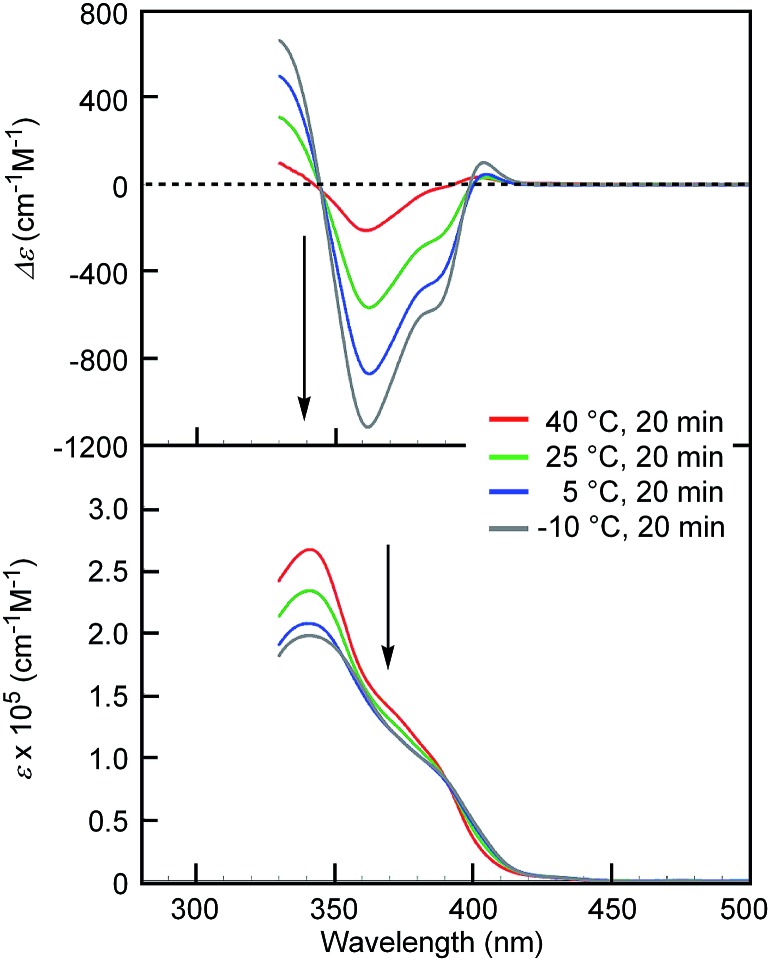
CD (top) and UV-Vis (bottom) spectra of [(*M*)-d-**4**]-C_12_-TEG in acetone (1 × 10^–5^ M) at different temperatures. Arrows show the changes upon cooling.

[(*M*)-d-**4**]-C_12_-TEG was soluble in non-aromatic polar solvents such as acetone and ethyl acetate even at a concentration of 1 × 10^–3^ M, at which conventional ethynylhelicene oligomers^5^ without TEG moieties were not soluble. The “ordinary” thermoresponse was observed in the polar solvents as well as in the aromatic solvent. A solution of [(*M*)-d-**4**]-C_12_-TEG in acetone (1 × 10^–5^ M) showed an increase in the CD intensity and a hypochromic shift in the UV-Vis spectrum upon cooling from 40 to –10 °C (Fig. 2), which indicated the formation of double helices upon cooling. Dimeric aggregate formation in acetone was confirmed by VPO studies (acetone, 45 °C, above 4 × 10^–3^ M) (ESI Fig. S3 and Table S3†). The CD analysis at a low concentration of 1 × 10^–5^ M (–10 °C) and at high concentrations of 5 × 10^–4^ M and 1 × 10^–3^ M (5 °C) converged on the same spectrum with the Δ*ε* of –1.1 × 10^3^ cm^–1^ M^–1^ at a wavelength of 360 nm (ESI Fig. S4†). It indicated that the spectrum was that of the equilibrium-shifted state to double-helices containing practically no random coils in the solution, namely S-double-helix state. The average diameter determined by dynamic light scattering (DLS) in acetone (1 × 10^–3^ M) at 5 °C was 5.2 nm (ESI Fig. S5a†), which was consistent with the VPO result showing dimeric aggregate formation, not polymolecular aggregate formation. At a lower concentration (1 × 10^–4^ M), particles of 3.8 nm and 0.76 nm in diameter were observed at 5 °C, which corresponded to double helices and random coils, respectively (ESI Fig. S5b†). The increase of larger particles and the decrease of smaller particles upon cooling from 25 and 40 °C are consistent with the formation of bimolecular double helices from monomeric random coils. In ethyl acetate, the “ordinary” thermoresponse was also observed in the CD and UV-Vis spectra (ethyl acetate, 1.0 × 10^–3^ M) (ESI Fig. S6†).

**Fig. 3:**
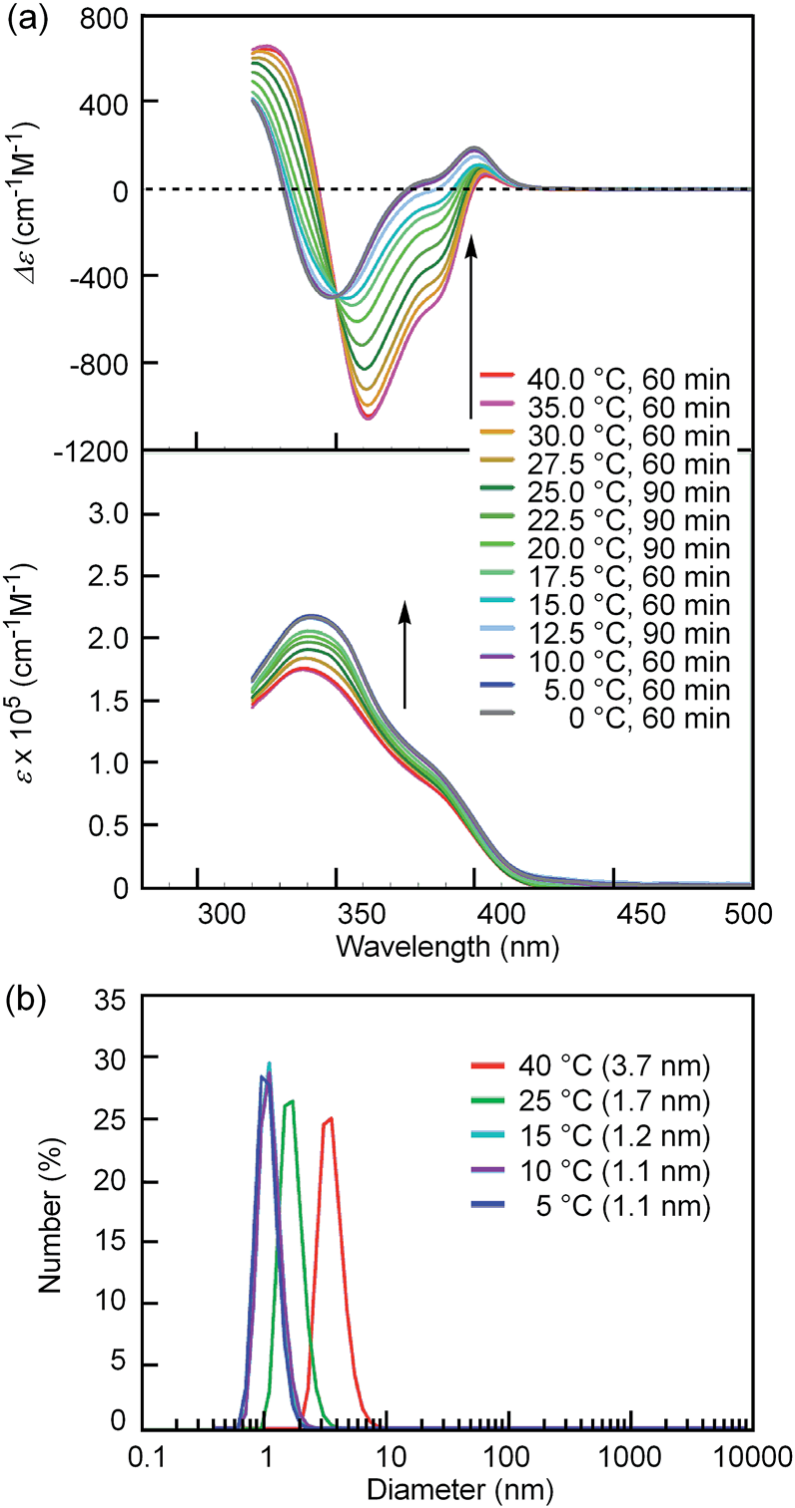
(a) CD (top) and UV-Vis (bottom) spectra and (b) number average size distributions of [(*M*)-d-**4**]-C_12_-TEG in acetone/water/triethylamine (1/2/1, 1 × 10^–5^ M) determined by DLS at different temperatures. Arrows show the changes upon cooling.

**Fig. 4:**
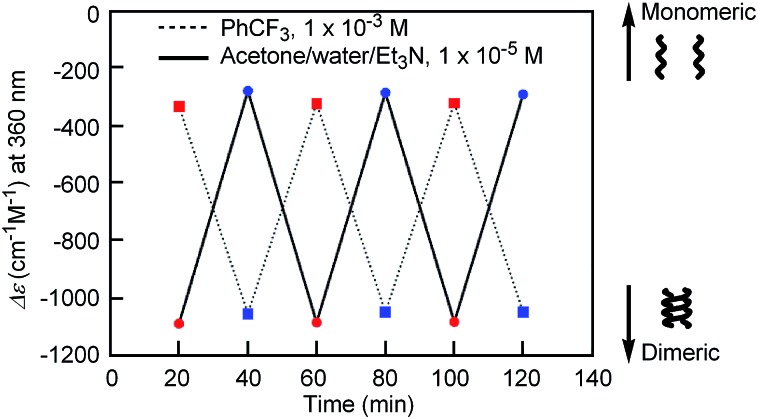
Plots of Δ*ε* at 360 nm of [(*M*)-d-**4**]-C_12_-TEG in acetone/water/triethylamine (1/2/1, 1 × 10^–5^ M) obtained by repeating the cycle of heating at 40 °C (red circles) and cooling at 5 °C (blue circles) for 20 min. Plots in trifluoromethylbenzene (1 × 10^–3^ M) obtained by repeating the cycle of heating at 60 °C (red squares) and cooling at 5 °C (blue squares) for 20 min are also shown.

Notably, the “inverse” thermoresponse of [(*M*)-d-**4**]-C_12_-TEG was observed in aqueous solvents. In a mixed solvent of acetone/water/triethylamine (1/2/1, v/v/v), intense Cotton effects (1 × 10^–5^ M) were observed at 40 °C (Fig. 3a), which coincided with that for the S-double-helix state in acetone (ESI Fig. S7†). Upon cooling, UV-Vis absorption increased and CD intensity decreased, which indicated disaggregation (Fig. 3a). An isosbestic point at 350 nm indicated an equilibrium between two states, the double helix and the random coil. The average diameters obtained by DLS in acetone/water/triethylamine (1/2/1, 1 × 10^–5^ M) were 3.7 nm, 1.7 nm, and 1.1 nm at 40 °C, 25 °C, and 5 °C, respectively (Fig. 3b).‡
‡The solubility of [(*M*)-d-**4**]-C_12_-TEG in the aqueous media was poor, and the apparent molecular weight was not obtained by a VPO measurement.The diameter at 40 °C coincided with that of the double helix in acetone (ESI Fig. S5a†), for which dimeric aggregate formation was confirmed by VPO (ESI Fig. S3†). The decrease in the diameter upon cooling is consistent with the disaggregation from a bimolecular double helix to a monomeric state. Thus, the “inverse” thermoresponse was observed for [(*M*)-d-**4**]-C_12_-TEG in acetone/water/triethylamine (1/2/1): the double helix was formed at 40 °C and the random coil at 5 °C. In addition, the “inverse” thermoresponse in this system was confirmed to be a molecular-level phenomenon in the dispersed state by DLS analysis; it was not caused by polymolecular aggregation. The spectra reversibly changed in response to changing temperature in a manner opposite to the “ordinary” thermoresponse (trifluoromethylbenzene, 1 × 10^–3^ M) (Fig. 4).

The “inverse” thermoresponse was also observed at different concentrations. Temperature-dependent changes in CD and UV-Vis spectra similar to those at 1 × 10^–5^ M were observed at 5 × 10^–6^ M and 1.5 × 10^–5^ M (Fig. S8a and b†). It is notable that dimeric aggregation occurred at these low concentrations. DLS analyses at these concentrations showed that the size of the aggregates were similar to those at 1 × 10^–5^ M (Fig. S9a and b†), and the “inverse” thermoresponse at the molecular level was confirmed. The “inverse” thermoresponse was observed at a higher concentration such as 3 × 10^–5^ M as well, although polymolecular aggregates partially formed below 20 °C, as indicated by CD and DLS (ESI Fig S8c and S9c†).

The CD spectra in acetone/water/triethylamine (1/2/1, 1 × 10^–5^ M) at 10 °C and 5 °C coincide, which show a convergence to a spectrum with a Δ*ε* of –3.1 × 10^2^ cm^–1^ M^–1^ at 360 nm. The CD spectra at different concentrations, 5 × 10^–6^ M and 1.5 × 10^–5^ M, also converge to the same spectra at 5 °C (ESI Fig. S8a and b†). In the following discussions, the spectrum in acetone/water/triethylamine (1/2/1, 1 × 10^–5^ M, 5 °C) with a Δ*ε* of –3.1 × 10^2^ cm^–1^ M^–1^ at 360 nm is defined as the S_aq_-random-coil state, which is the equilibrium-shifted state to random coils in aqueous media practically containing no double helices. It was noted that the CD spectra of the random-coil state in organic solvents (ESI Fig. S1a†) and the S_aq_-random-coil state in aqueous solvents (Fig. 3a) are slightly different, which may be due to a specific conformation of TEG moieties in aqueous media.^9^

The “inverse” thermoresponse also occurred when the amount of acetone was changed to 0.6/2/1 and 0.8/2/1, keeping the concentration of [(*M*)-d-**4**]-C_12_-TEG at 1 × 10^–5^ M (ESI Fig. S10†).

Note that [(*M*)-d-**4**]-C_12_-TEG provided an unprecedented example of dimeric aggregation with the “inverse” thermoresponse, which is in contrast to the other known synthetic double helices that exhibit the “ordinary” thermoresponse.^4^,^5^

An examination of the solvents revealed the critical roles of water and triethylamine. In a mixed solvent of acetone/triethylamine (3/1, 1 × 10^–5^ M), the “ordinary” thermoresponse was observed: CD and UV-Vis spectra of the random-coil state were obtained at 40 °C and 25 °C. The spectra changed at 5 °C, and those of partial double helices were obtained upon cooling to –10 °C (ESI Fig. S11a†). The presence of molecular-level aggregates but not polymolecular aggregates was confirmed by DLS, which showed average diameters from 0.84 to 0.88 nm at 25 °C (ESI Fig. S12†). In acetone/water (3/1, 1 × 10^–5^ M), the system was opaque, spectra of the random-coil state were obtained by CD at 40, 25, 5, and –10 °C (Fig. S11b),§
§Acetone/water (3/1, 1 × 10^–5^ M) systems were opaque, and the formation of polymolecular assemblies were suggested. Studies of the assemblies are ongoing. which may be due to the formation of polymolecular aggregates of random coils. The results indicated that both water and triethylamine are necessary for the “inverse” thermoresponse of [(*M*)-d-**4**]-C_12_-TEG.

Variable-temperature ^1^H NMR studies (acetone-*d*_6_/D_2_O/triethylamine-*d*_15_, 1/2/1, 2 × 10^–4^ M) were conducted to obtain insight into the thermoresponse of [(*M*)-d-**4**]-C_12_-TEG in aqueous solvents. Broad proton signals of the terminal TEG groups were observed between *δ* 2.4–3.5 at 25 °C (ESI Fig. S13†). When the temperature was increased to 40 °C, the signals of the TEG groups became sharper and increased in intensity. An upfield shift of the chemical shifts of HDO signals was also observed. These results are consistent with the reported hydration/dehydration of poly(ethylene glycol) (PEG) groups.^10^,^11^

Thermodynamic parameters of the double-helix formation with the “inverse” thermoresponse were experimentally determined using equilibrium constants *K* (Table S4†) obtained from the CD Δ*ε* values at 360 nm in acetone/water/triethylamine (1/2/1, 1 × 10^–5^ M), Δ*H* = +2.4 × 10^2^ kJ mol^–1^ and Δ*S* = +9.2 × 10^2^ J mol^–1^ K^–1^ (ESI Fig. S14†). Note that both Δ*H* and Δ*S* are positive and large. This contrasted with the “ordinary” thermoresponse of the dimeric aggregation of molecules giving negative Δ*H* and Δ*S* values,^1^,2b,^3^,4c,d including conventional ethynylhelicene oligomers in organic solvents.5*b* The positive Δ*H* and Δ*S* consequently induced an increase in the dimerization constant *K* upon heating according to the equation *R* ln *K* = –Δ*H*/*T* + Δ*S*, which appeared as the “inverse” thermoresponse (Fig. 1b). The result validated the hypothesized discussion in the introduction.

A significant change in the double helix/random coil ratio was observed in response to small temperature changes (Table S4†). For example, the double helix/random coil ratios in acetone/water/triethylamine (1/2/1, 1 × 10^–5^ M) at 40 °C and 10 °C were estimated to be 90%/10% and 3%/97%, respectively. The large Δ*H* resulted in a substantial “inverse” thermoresponse.

The positive Δ*H* and Δ*S* of the “inverse” thermoresponse are counter intuitive in the dimeric molecular aggregation but can be explained by the hydration/dehydration of the PEG moiety. Dehydration upon heating makes the PEG moiety hydrophobic, which reduces the thermodynamic stability of random coils in aqueous solvents (Δ*H* > 0) and promotes dimeric aggregation. When the PEG moieties are dehydrated, Δ*S* increases and overcomes the decrease in Δ*S* resulting from dimeric aggregation. It is known that PEG and oligo(ethylene glycol) (OEG) are hydrated in water below the temperature defined as the lower critical solution temperature (LSCT), and that dehydration upon heating enhances hydrophobic interactions. Then, polymolecular aggregation and precipitation occur to reduce the molecular surface area exposed to water.^11^–^14^ Similar heat-induced aggregation^15^ and self-assembly^16^ related to the hydration/dehydration of biological peptide and protein molecules have also been reported.

**Fig. 5:**
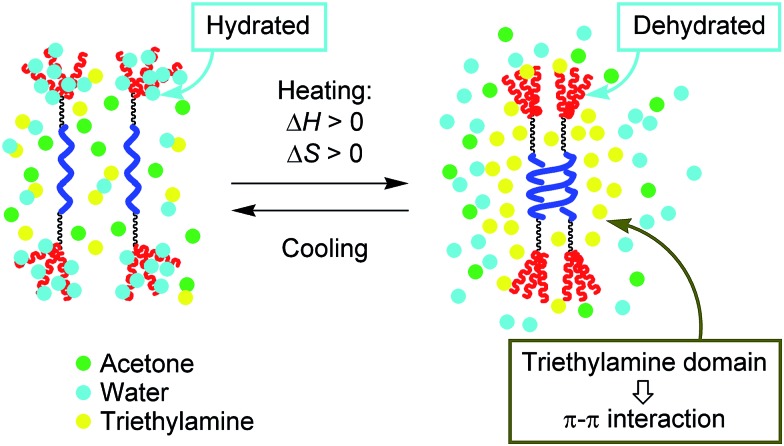
Schematic representation of the explanation for the “inverse” thermoresponse of [(*M*)-d-**4**]-C_12_-TEG in acetone/water/triethylamine (1/2/1).

It should be noted here that [(*M*)-d-**4**]-C_12_-TEG formed dimeric aggregates upon heating, not polymolecular aggregates, which is another unusual aspect of the “inverse” thermoresponse phenomenon in this system. Such dimeric aggregate formation of synthetic molecules has not been reported. The result reminds us of peptides and enzymes,^15^,^17^ which control their activities by forming dimeric aggregates upon heating and disaggregating to monomers upon cooling. In our system, triethylamine is considered to play a crucial role in the formation of dimeric aggregates of [(*M*)-d-**4**]-C_12_-TEG. Triethylamine and water are known to form hydrogen-bonds at low temperatures, and microscopic phase separation occurs upon heating.^18^ The resulting triethylamine domains incorporate dehydrated [(*M*)-d-**4**]-C_12_-TEG molecules (Fig. 5). Fewer polar environments made by organic solvents can promote the dimeric aggregate formation of ethynylhelicene oligomer moieties, which is enthalpically driven by π–π interactions, and does not induce polymolecular aggregation as a result of the hydrophobic interactions. It should be emphasized again that the “inverse” thermoresponse in dimeric aggregate formation shown in this study is a molecular-level phenomenon in the dispersed solution state and is different from the phenomenon in which polymolecular aggregates are formed by hydrophobic interactions above the LCST.

## Materials and methods

### Synthesis of [(*M*)-d-**4**]-C_12_-TEG

Under an argon atmosphere, a mixture of **4** (45.0 mg, 0.0451 mmol), tris(dibenzylideneacetone)dipalladium(0) chloroform adduct (0.82 mg, 0.753 μmol), cuprous iodide (1.72 mg, 9.03 μmol), tris(2,4,6-trimethylphenyl)phosphine (1.75 mg, 4.52 μmol), triphenylphosphine (1.18 mg, 4.52 μmol), tetrabutylammonium iodide (22.2 mg, 0.0602 mmol), triethylamine (0.1 mL) and *N*,*N*-dimethylformamide (1.0 mL) was freeze-evacuated four times in flask A. In flask B, a mixture of ethynylhelicene tetramer (*M*)-d-**4H**5*e* (30.0 mg, 0.0151 mmol) in THF (1.0 mL) was freeze-evacuated four times, and the mixture was slowly added to flask A. The mixture was stirred at 45 °C for 20 min. The reaction was quenched by adding saturated aqueous ammonium chloride, and the organic materials were extracted with ethyl acetate. The organic layer was washed with brine, and dried over sodium sulfate. The solvents were evaporated under reduced pressure, and separation by silica gel chromatography and recycling GPC gave [(*M*)-d-**4**]-C_12_-TEG as a yellow amber solid (42.7 mg, 0.0115 mmol, 72%). *M*_p_: 59–61 °C (chloroform); [α]27D = –1631 (c 0.37, trifluoromethylbenzene); ^1^H NMR (400 MHz, CDCl_3_): *δ* 0.86 (9H, t, *J* = 6.8 Hz), 1.25–1.52 (74H, m), 1.72–1.89 (14H, m), 1.97 (12H, s), 2.00 (12H, s), 3.37 (18H, s), 3.52–3.55 (12H, m), 3.62–3.68 (24H, m), 3.70–3.75 (12H, m), 3.80 (4H, t, *J* = 5.2 Hz), 3.87 (8H, t, *J* = 5.0 Hz), 4.01 (4H, t, *J* = 6.6 Hz), 4.18–4.23 (12H, m), 4.28 (4H, t, *J* = 6.8 Hz), 4.41–4.45 (6H, m), 6.94 (4H, dt, *J* = 8.8, 1.8 Hz), 7.29 (4H, s), 7.46–7.52 (8H, m), 7.64 (4H, dt, *J* = 8.8, 1.8 Hz), 7.66–7.77 (8H, m), 8.06 (2H, s), 8.12 (2H, s), 8.16 (4H, s), 8.21 (2H, t, *J* = 1.6 Hz) 8.22 (1H, t, *J* = 1.6 Hz), 8.36–8.38 (6H, m), 8.52–8.58 (8H, m); ^13^C NMR (100 MHz, CDCl_3_): *δ* 14.1, 22.6, 23.2, 25.96, 26.02, 28.7, 29.2, 29.3, 29.4, 29.5, 29.6, 31.8, 58.97, 59.00, 65.2, 65.8, 68.1, 68.8, 69.6, 70.49, 70.52, 70.6, 70.8, 71.9, 72.4, 86.2, 89.2, 89.3, 89.4, 92.8, 92.97, 92.99, 95.1, 109.0, 114.6, 115.1, 119.6, 119.816, 119.818, 120.9, 123.5, 123.6, 123.7, 124.2, 124.28, 124.33, 125.3, 126.2, 126.7, 126.8, 127.0, 128.82, 128.84, 129.1, 129.2, 129.3, 129.8, 129.88, 129.92, 130.9, 131.01, 131.03, 131.2, 131.4, 132.0, 132.2, 132.4, 133.2, 136.76, 136.80, 136.9, 138.3, 142.5, 152.2, 159.4, 165.4, 166.1; IR (KBr): 2924, 1717, 1244, 1111 cm^–1^; UV-Vis (S-random-coil state: CHCl_3_, 5 × 10^–4^ M, 40 °C): *λ*_max_ (*ε*) 344 nm (3.1 × 10^5^ cm^–1^ M^–1^); UV-Vis (S-double-helix state: acetone, 1 × 10^–3^ M, 5 °C): *λ*_max_ (*ε*) 340 nm (2.0 × 10^5^ cm^–1^ M^–1^); CD (S-random-coil state: CHCl_3_, 5 × 10^–4^ M, 40 °C): *λ* (Δ*ε*) 296 nm (+51 cm^–1^ M^–1^), 341 nm (–76 cm^–1^ M^–1^), 389 nm (+189 cm^–1^ M^–1^); CD (S-double-helix state: acetone, 1 × 10^–3^ M, 5 °C): *λ* (Δ*ε*) 325 nm (+679 cm^–1^ M^–1^), 362 nm (–1133 cm^–1^ M^–1^); MALDI-TOF MS (*m*/*z*): [M + Na]^+^ calcd for C_239_H_278_O_36_Na, 3747.0; found, 3746.3; [M + K]^+^ calcd for C_239_H_278_O_36_K, 3763.0; found, 3763.0; analysis (calcd, found for C_239_H_278_O_36_): C (77.03, 76.90), H (7.52, 7.54).

## Conclusions

In summary, [(*M*)-d-**4**]-C_12_-TEG, an ethynylhelicene oligomer with six tri(ethylene glycol) moieties at its termini, was synthesized. [(*M*)-d-**4**]-C_12_-TEG formed double helices in aromatic solvents, polar non-aromatic solvents, and the aqueous solution of acetone/water/triethylamine. [(*M*)-d-**4**]-C_12_-TEG exhibited the “inverse” thermoresponse in acetone/water/triethylamine (1/2/1): [(*M*)-d-**4**]-C_12_-TEG aggregated and formed double helices upon heating and disaggregated to random coils upon cooling. The double helix/random coil ratio sharply and reversibly changed in response to thermal stimuli. This is an unprecedented molecular-level “inverse” thermoresponse, in which dimeric aggregates but not polymolecular aggregates are formed. Positive and large Δ*H* and Δ*S* values in the aggregation process were determined, which were explained by the dehydration of terminal TEG groups upon heating and the formation of triethylamine domains that promoted double-helix formation by π–π interactions.

## Note added after first publication

This article replaces the version published on 22nd February 2016, which contained errors in the grant numbers reported in the acknowledgements section.

## Supplementary Material

Supplementary information

## References

[cit1] Saito N., Terakawa R., Yamaguchi M. (2014). Chem.–Eur. J..

[cit2] Catalán J. (2010). J. Phys. Chem. A.

[cit3] Sun H., Ye K., Wang C., Qi H., Li F., Wang Y. (2006). J. Phys. Chem. A.

[cit4] Yamada H., Wu Z.-Q., Furusho Y., Yashima E. (2012). J. Am. Chem. Soc..

[cit5] Saito N., Terakawa R., Shigeno M., Amemiya R., Yamaguchi M. (2011). J. Org. Chem..

[cit6] (a) Cantor C. R. and Schmmel P. R., Biophysical Chemistry Part III: The Behavior of Biological Macromolecules, W. H. Freeman and Co., San Francisco, 1980.

[cit7] The “inverse” thermoresponse of a synthetic molecule was suggested by the shift of ^1^H NMR signals, but the ratio of monomer and dimer was not clarified: Brück A. Kilway K. V., Tetrahedron, 2001, 57 , 7263 –7268 , . In ref. 3*a*, only one molecule gave a positive Δ*H* in a very limited temperature range .

[cit8] Münzenberg C., Rossi A., Feldman K., Fiolka R., Stermmer A., Kita-Tokarczyk K., Meier W., Sakamoto J., Lukin O., Schülter A. D. (2008). Chem.–Eur. J..

[cit9] Muraoka T., Adachi K., Ui M., Kawasaki S., Sadhukhan N., Obara H., Tochio H., Shirakawa M., Kinbara K. (2013). Angew. Chem., Int. Ed..

[cit10] Shikata T., Okuzono M., Sugimoto N. (2013). Macromolecules.

[cit11] Zhang C., Peng H., Whittaker A. K. (2014). J. Polym. Sci., Part A: Polym. Chem..

[cit12] Liao Y., Dong C.-M. (2012). J. Polym. Sci., Part A: Polym. Chem..

[cit13] Chi X., Xue M. (2014). Chem. Commun..

[cit14] Dong S., Zheng B., Yao Y., Han C., Yuan J., Antonietti M., Huang F. (2013). Adv. Mater..

[cit15] Ruan K., Weber G. (1988). Biochemistry.

[cit16] Lauffer M. A., Ansevin A. T., Cartwright T. E., Brinton C. C. (1958). Nature.

[cit17] Isohashi H., Nakanishi Y., Sakamoto Y. (1983). Eur. J. Biochem..

[cit18] Kajimoto S., Yoshii N., Hobley J., Fukumura H., Okazaki S. (2007). Chem. Phys. Lett..

