# Supplementary material for: “Inverse” thermoresponse: heat-induced double-helix formation of an ethynylhelicene oligomer with tri(ethylene glycol) termini
Source: Chem Sci. 2016 Feb 12;7(6):3574–80. doi: 10.1039/c5sc04959h (PMC6007355; doi:10.1039/c5sc04959h)
Supplement: Supplementary file 1 [file SC-007-C5SC04959H-s001.pdf]

Electronic Supplementary Material (ESI) for Chemical Science.
This journal is © The Royal Society of Chemistry 2016

"Inverse" Thermoresponse: Heat-Induced Double-Helix Formation of An Ethynylhelicene Oligomer with Tri(Ethylene Glycol) Termini

Nozomi Saito,[a, b] Higashi Kobayashi,[a] and Masahiko Yamaguchi*[a]

[a] Dr. N. Saito,H. Kobayashi, and Prof. Dr. M. Yamaguchi

Department of Organic Chemistry, Graduate School of Pharmaceutical Sciences, Tohoku University, Aoba, Sendai 980-8578, Japan

[b] Dr. N. Saito

Tohoku University Frontier Research Institute for Interdisciplinary Sciences, Aoba, Sendai 980-8578, Japan

*E-mail: yama@m.tohoku.ac.jp

FAX: +81-022-795-6811

Electronic Supplementary Information

Table of Contents

General method……S2
Procedure for CD and UV-Vis measurement……S2
Procedure for VPO……S2
Procedure for DLS Analyses…… S3
Synthesis of [(*M*)-D-4]-C_{12}-TEG and compound characterization…… S4-6
CD, UV-Vis, VPO, and DLS studies of [(*M*)-D-4]-C_{12}-TEG in organic solvents…… S7-10
CD, UV-Vis, and DLS studies of [(*M*)-D-4]-C_{12}-TEG in acetone/water/triethylamine…. S11-13
CD, UV-Vis, and DLS studies of [(*M*)-D-4]-C_{12}-TEG in acetone/triethylamine…… S14
Variable-temperature ^{1}H-NMR study of [(*M*)-D-4]-C_{12}-TEG in acetone/water/triethylamine...... S15
Double helix/random coil ratio…… S16
Thermodynamic parameters of double-helix formation of [(*M*)-D-4]-C_{12}-TEG in acetone/water/triethylamine…… S17-18
^{1}H and ^{13}C Spectra……S19-27
References……S28

< General method >

Melting points were determined with a Yanaco micro melting point apparatus without correction. Elemental analyses were conducted with a Yanaco CHN CORDER MT-6. Optical rotations were measured on a JASCO DIP-340 digital polarimeter. IR spectra were measured on a JASCO FT/IR-400 spectrophotometer. UV-Vis spectra were measured on a BECKMAN DU 640 or a JASCO J-720 spectropolarimeter. ^{1}H NMR (400 MHz) spectra were recorded on a Varian Vnmr J2.2C with tetramethylsilane as an internal standard. ^{13}C NMR spectra were recorded on a Varian Vnmr J2.2C (100 MHz) or a JEOL JNM-ECA 600 (151 MHz), and were referenced to the residual solvents $CDCl_3$ (δ 77.0). Chemical shifts are expressed in parts per million (ppm, δ). The abbreviations of signal patterns are as follows: s, singlet; d, doublet; t, triplet; q, quartet. Low- and High- resolution mass spectra were recorded on a JEOL JMS-DX-303, a JMS-AX-500, or a JEOL JMS-700. MALDI-TOF MS spectra were recorded on a Shimadzu AXIMA Assurance using α-cyano-4-hydroxycinnamic acid as the matrix. CD spectra were measured on a JASCO J-720 spectropolarimeter. Vapor pressure osmometry (VPO) was conducted with KNAUER K-7000 molecular weight apparatus using benzil as a standard. Gel permeation chromatography (GPC) was conducted with Recycling Preparative HPLC LC-908 or LC-918 (Japan Analytical Industry, Co. Ltd.). CD and UV-Vis spectra were recorded using distilled or spectrophotomeric grade purchased solvents, and LC/MS grade purchased ultra pure water. Dynamic light scattering (DLS) studies were conducted using a Zetasizer Nano S90 with the scattering angle of 173°. Viscosities of solvent mixtures were measured using SV-10 (A&D Instruments Ltd.). The ratio of solvent mixture is shown in volume/volume.

< Procedure for CD and UV-Vis measurement >

[(*M*)-D-**4**]-C_{12}-TEG was dissolved in a solvent, heated at 60 °C or 40 °C, and cooled to different temperatures for CD and UV-Vis analysis. Spectra at a steady state at each temperature were obtained by monitoring the spectra every 10 min until convergence is confirmed.

< **Procedure for VPO** >

The VPO measurements were conducted after keeping the solution for longer than 30 min in syringes at the measurement temperature. The results shown are the average of five obtained results.

< **Procedure for DLS Analyses** >

Dynamic light scattering (DLS) studies were conducted with the scattering angle of 173°. The results shown are the average of ten obtained results. Viscosities and refractive indexes of the solvents used for the DLS analysis are shown below (Table S1).

Table S1.

Solvent	Temp.	Viscosity (mPa·S)	Refractive index
acetone	40 °C	0.2758[(a)]	1.356 [(a)]
	25 °C	0.3111[(a)]	1.356 [(a)]
	5 °C	0.3729[(a)]	1.356 [(a)]
acetone/water/triethylamine (1/2/1)	40 °C	0.7929[(b)]	1.356[(c)]
	25 °C	1.3263[(b)]	1.356 [(c)]
	15 °C	2.2692[(b)]	1.356 [(c)]
	10 °C	2.7229[(b)]	1.356 [(c)]
	5 °C	3.1474[(b)]	1.356 [(c)]
acetone/triethylamine (3/1)	40 °C	0.2758[(d)]	1.356[(d)]
	25 °C	0.3111[(d)]	1.356 [(d)]
	5 °C	0.3729[(d)]	1.356 [(d)]

[(a)] Values for acetone installed in the Zetasizer Nano S90 were used.
[(b)] Experimentally obtained.
[(c)] Values for acetone/ water (1/1) installed in the Zetasizer Nano S90 were used.
[(d)] Values for acetone installed in the Zetasizer Nano S90 were used.

< Synthesis of [(*M*)-D-4]-C_{12}-TEG and compound characterization>

Scheme S1.

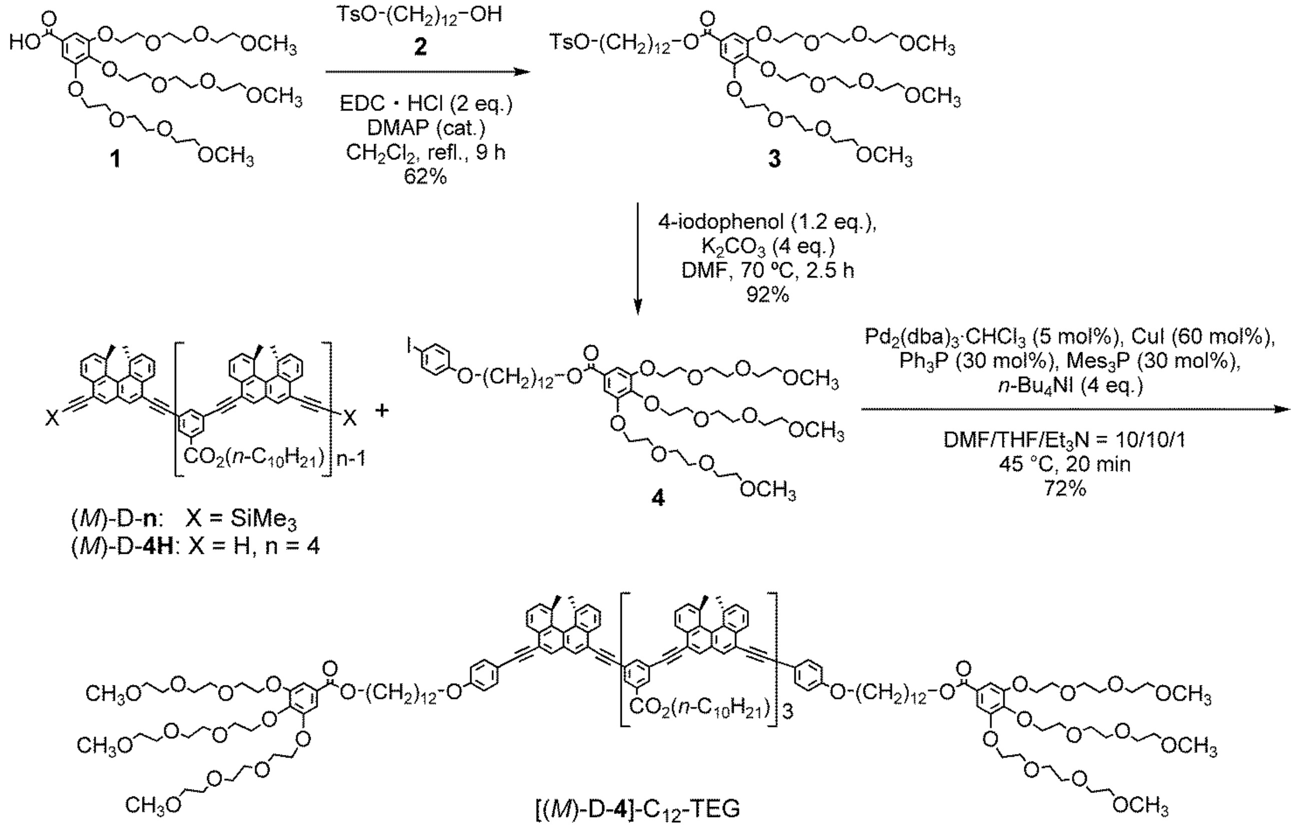

3,4,5-Tris{2-[2-(2-methoxyethoxy)ethoxy]ethoxy} benzoic acid 12-{[(4-methylphenyl)sulfonyl] oxy}dodecyl ester 3. Under an argon atmosphere, a mixture of 3,4,5-tris{2-[2-(2-methoxyethoxy)ethoxy]ethoxy} benzoic acid **1**[S1] (200 mg, 0.329 mmol), 1,12-dodecanediol mono(4-methlybenzenesulfonate) **2** (117 mg, 0.329 mmol), *N*-(3-dimethylaminopropyl)-*N*'-ethylcarbodiimide hydrochloride (92 mg, 0.482 mmol), 4-(dimethylamino)pyridine (cat.) and dichloromethane (12.5 mL) was heated under reflux for 9 h. After cooling to room temperature, the solvent was evaporated under reduced pressure, and separation by silica gel chromatography gave **3** as colorless oil (192.7 mg, 0.203 mmol, 62%). LRMS (EI, 70 eV) *m/z*: 947 ($[M]^+$, 0.7%), 775 ($[M^+{-}HOSO_2C_6H_4CH_3]^+$, 13%), 147 ($[(CH_2CH_2O)_3CH_3]^+$, 100%), 103 ($[(CH_2CH_2O)_2CH_3]^+$, 21%), 59 ($[CH_2CH_2OCH_3]^+$, 21%). HRMS *m/z* Calcd for $C_{47}H_{78}O_{17}S$: 946.4960. Found: 946.4960. IR (neat) 2925, 1713, 1429, 1359, 1333, 1177, 1111 cm^{-1}. ^1H NMR (400 MHz, $CDCl_3$) δ 1.22–1.43 (16H, m), 1.63 (2H, quint, *J* = 6.9 Hz), 1.75 (2H, quint, *J* = 7.1 Hz), 2.45 (3H, s), 3.37 (9H, s), 3.53–3.56 (6H, m) 3.62–3.69 (12H, m), 3.71–3.75 (6H, m), 3.80 (2H, t, *J* = 5.0 Hz), 3.87 (4H, t, *J* = 5.0 Hz), 4.02 (2H, t, *J* = 6.6 Hz), 4.18–4.23 (6H, m), 4.28 (2H, t, *J* = 6.8 Hz), 7.29 (2H, s) 7.34 (2H, d, *J* = 8.0 Hz), 7.79 (2H, d, *J* = 8.0 Hz). ^{13}C NMR (100 MHz, $CDCl_3$) δ 21.5, 25.2, 25.8, 28.6, 28.7, 28.8, 29.1, 29.2, 29.3, 29.37, 29.38, 58.87, 58.89, 65.1, 68.7, 69.5, 70.40, 70.42, 70.5, 70.7, 71.8, 72.3, 108.9, 125.2, 127.7, 129.7, 133.1, 142.4, 144.5, 152.1, 166.0.

3,4,5-Tris{2-[2-(2-methoxyethoxy)ethoxy]ethoxy} benzoic acid 12-(4-iodophenoxy)dodecyl ester 4. Under an argon atmosphere, a mixture of **3** (829 mg, 0.876 mmol), 4-iodophenol (231 mg, 1.05 mmol), potassium carbonate (484 mg, 3.51 mmol) and *N*,*N*-dimethylformamide (30 mL) was stirred at 70 °C for 2.5 h. The mixture was cooled to room temperature, and saturated aqueous ammonium chloride was added. The organic materials were extracted with ethyl acetate. The organic layer was washed with brine, and dried over sodium sulfate. The solvent was evaporated under reduced pressure, and separation by silica gel chromatography gave **4** as pale yellow oil (802 mg, 0.806 mmol, 92%). LRMS (EI, 70 eV) *m/z*: 994 ($[M]^+$, 44%), 848 ($[M{-}CHCH_2O(CH_2CH_2O)_2CH_3]^+$, 26%), 147 ($[(CH_2CH_2O)_3CH_3]^+$, 100%), 103 ($[(CH_2CH_2O)_2CH_3]^+$, 77%), 59 ($[CH_2CH_2OCH_3]^+$, 78%). HRMS *m/z* Calcd for $C_{46}H_{75}O_{15}I$: 994.4151. Found: 994.4150. IR (neat) 2925, 1714, 1487, 1429, 1331, 1243, 1110 cm^{-1}. ^1H NMR (400 MHz, $CDCl_3$) δ 1.25−1.43 (16H, m), 1.75 (4H, quint, *J* = 7.1 Hz), 3.37 (9H, s), 3.53−3.55 (6H, m), 3.62−3.69 (12H, m), 3.71−3.75 (6H, m), 3.80 (2H, dd, *J* = 5.8, 4.6 Hz), 3.86−3.92 (6H, m), 4.19−4.24 (6H, m), 4.28 (2H, t, *J* = 6.8 Hz), 6.67 (2H, dd, *J* = 7.2, 2.6 Hz), 7.30 (2H, s), 7.53 (2H, dd, *J* = 7.2, 2.6 Hz). ^{13}C NMR (100 MHz, $CDCl_3$) δ 25.79, 25.80, 28.5, 28.9, 29.1, 29.2, 29.3, 29.4, 58.8, 65.0, 67.9, 68.7, 69.4, 70.3, 70.4, 70.5, 70.6, 71.7, 72.2, 82.2, 108.9, 116.7, 125.1, 137.9, 142.4, 152.1, 158.8, 165.9.

(*M*)-Ethynylhelicene tetramer with PEG terminal groups, [(*M*)-D-4]-C_{12}-TEG. Under an argon atmosphere, a mixture of **4** (45.0 mg, 0.0451 mmol), tris(dibenzylideneacetone)dipalladium(0) chloroform adduct (0.82 mg, 0.753 μmol), cuprous iodide (1.72 mg, 9.03 μmol), tris(2,4,6-trimethylphenyl)phosphine (1.75 mg, 4.52 μmol), triphenylphosphine (1.18 mg, 4.52 μmol), tetrabutylammonium iodide (22.2 mg, 0.0602 mmol), triethylamine (0.1 mL) and *N,N*-dimethylformamide (1.0 mL) was freeze-evacuated four times in flask A. In flask B, a mixture of ethynylhelicene tetramer (*M*)-D-**4H**[S2] (30.0 mg, 0.0151 mmol) in THF (1.0 mL) was freeze-evacuated four times, and the mixture was slowly added to flask A. The mixture was stirred at 45 °C for 20 min. The reaction was quenched by adding saturated aqueous ammonium chloride, and the organic materials were extracted with ethyl acetate. The organic layer was washed with brine, and dried over sodium sulfate. The solvents were evaporated under reduced pressure, and separation by silica gel chromatography and recycling GPC gave [(*M*)-D-**4**]-C_{12}-TEG as yellow amber solid (42.7 mg, 0.0115 mmol, 72%). Mp 59−61 °C (chloroform). $[\alpha]_D^{27}$ −1631 (c 0.37, trifluoromethylbenzene). MALDI-TOF MS *m/z* Calcd for $C_{239}H_{278}O_{36}$: 3724.0. Found: 3746.3 $[M+Na]^+$, 3763.0 $[M+K]^+$. UV-Vis (S-random-coil state: $CHCl_3$, 5×10^{-4} M, 40 °C) λ_{max} (ε) 344 nm (3.1×10^5). UV-Vis (S-double-helix state: acetone, 1×10^{-3} M, 5 °C) λ_{max} (ε) 340 nm (2.0×10^5). CD (S-random-coil state: $CHCl_3$, 5×10^{-4} M, 40 °C) λ ($\Delta\varepsilon$) 296 nm (+51), 341 nm (−76), 389 nm (+189). CD (S-double-helix state: acetone, 1×10^{-3} M, 5 °C) λ ($\Delta\varepsilon$) 325 nm (+679), 362 nm (−1133). IR (KBr) 2924, 1717, 1244, 1111 cm^{-1}. Anal. ($C_{239}H_{278}O_{36}$) Calcd: C, 77.03; H, 7.52. Found: C, 76.90; H, 7.54. ^1H NMR (400 MHz, $CDCl_3$) δ 0.86 (9H, t, *J* = 6.8 Hz), 1.25-1.52 (74H, m), 1.72-1.89 (14H, m), 1.97 (12H, s), 2.00 (12H, s), 3.37 (18H, s), 3.52-3.55 (12H, m), 3.62-3.68 (24H, m), 3.70-3.75 (12H, m), 3.80 (4H, t, *J* = 5.2 Hz), 3.87 (8H, t, *J* = 5.0 Hz), 4.01 (4H, t, *J* = 6.6 Hz), 4.18-4.23 (12H, m), 4.28 (4H, t, *J* = 6.8 Hz), 4.41-4.45 (6H, m), 6.94 (4H, dt, *J* = 8.8, 1.8 Hz),

7.29 (4H, s), 7.46-7.52 (8H, m), 7.64 (4H, dt, *J* = 8.8, 1.8 Hz), 7.66-7.77 (8H, m), 8.06 (2H, s), 8.12 (2H, s), 8.16 (4H, s), 8.21 (2H, t, *J* = 1.6 Hz) 8.22 (1H, t, *J* = 1.6 Hz), 8.36-8.38 (6H, m), 8.52-8.58 (8H, m). ^{13}C NMR (100 MHz, $CDCl_3$) δ 14.1, 22.6, 23.2, 25.96, 26.02, 28.7, 29.2, 29.3, 29.4, 29.5, 29.6, 31.8, 58.97, 59.00, 65.2, 65.8, 68.1, 68.8, 69.6, 70.49, 70.52, 70.6, 70.8, 71.9, 72.4, 86.2, 89.2, 89.3, 89.4, 92.8, 92.97, 92.99, 95.1, 109.0, 114.6, 115.1, 119.6, 119.816, 119.818, 120.9, 123.5, 123.6, 123.7, 124.2, 124.28, 124.33, 125.3, 126.2, 126.7, 126.8, 127.0, 128.82, 128.84, 129.1, 129.2, 129.3, 129.8, 129.88, 129.92, 130.9, 131.01, 131.03, 131.2, 131.4, 132.0, 132.2, 132.4, 133.2, 136.76, 136.80, 136.9, 138.3, 142.5, 152.2, 159.4, 165.4, 166.1.

< CD, UV-Vis, VPO, and DLS studies of [(*M*)-D-4]-C_{12}-TEG in organic solvents >

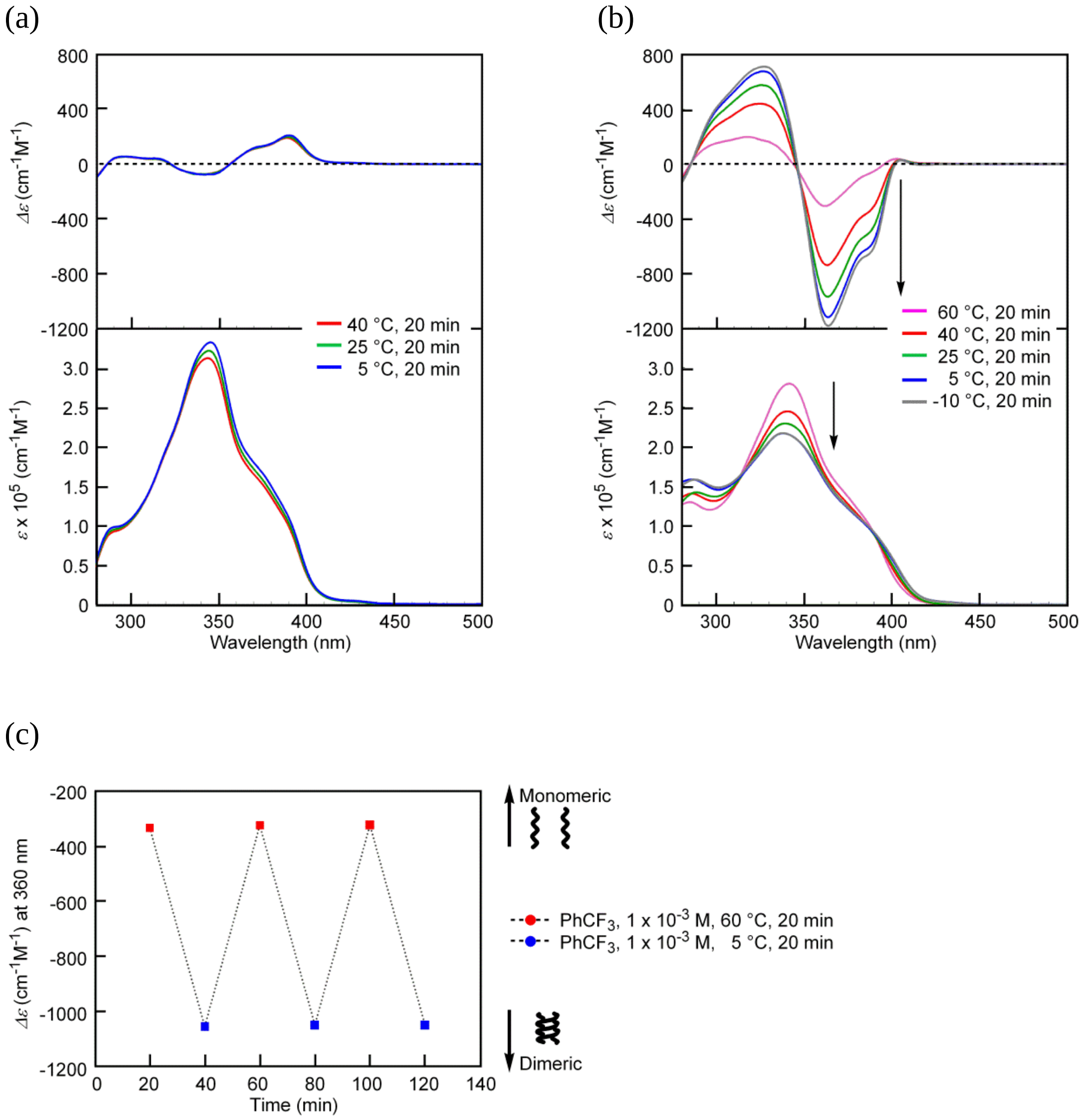

Figure S1. CD (top) and UV-Vis (bottom) spectra of [(*M*)-D-**4**]-C_{12}-TEG in (a) chloroform (5×10^{-4} M) and (b) trifluoromethylbenzene (1×10^{-3} M) at different temperatures. Arrows show the changes upon cooling. (c) Plots of $\Delta\varepsilon$ at 360 nm of [(*M*)-D-**4**]-C_{12}-TEG in trifluoromethylbenzene (1×10^{-3} M) obtained by repeating the cycle of heating at 60 °C and cooling at 5 °C for 20 min.

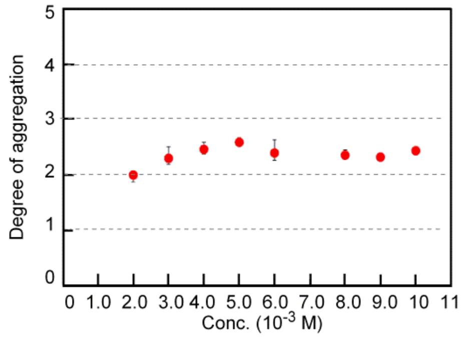

Figure S2. VPO analysis of [(*M*)-D-**4**]-C_{12}-TEG in trifluoromethylbenzene (40 °C) at different concentrations. Red circles represent the averages of obtained five measurements at each concentration, and the vertical black lines represent the range of results obtained.

Table S2. The apparent molecular weight of [(*M*)-D-**4**]-C_{12}-TEG obtained by VPO in trifluoromethylbenzene (40 °C) at different concentrations.

Concentration (10^{-3} M)	Apparent molecular weight
2.0	7.3×10^3
3.0	8.5×10^3
4.0	9.1×10^3
5.0	9.6×10^3
6.0	8.9×10^3
8.0	8.7×10^3
9.0	8.6×10^3
10	9.0×10^3

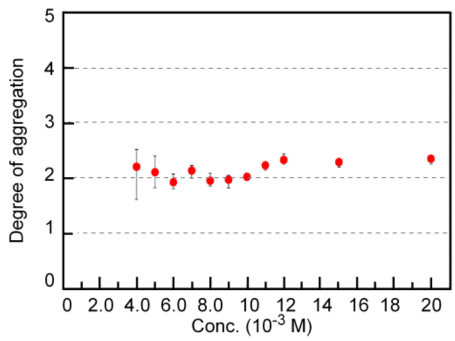

Figure S3. VPO analysis of [(*M*)-D-**4**]-C_{12}-TEG in acetone (45 °C) at different concentrations. Red circles represent the averages of obtained five measurements at each concentration, and the vertical black lines represent the range of results obtained.

Table S3. The apparent molecular weight of [(*M*)-D-**4**]-C_{12}-TEG obtained by VPO in acetone (45 °C) at different concentrations.

Concentration (10^{-3} M)	Apparent molecular weight
4.0	8.2×10^3
5.0	7.8×10^3
6.0	7.2×10^3
7.0	7.9×10^3
8.0	7.3×10^3
9.0	7.3×10^3
10	7.5×10^3
11	8.3×10^3
12	8.7×10^3
15	8.5×10^3
20	8.7×10^3

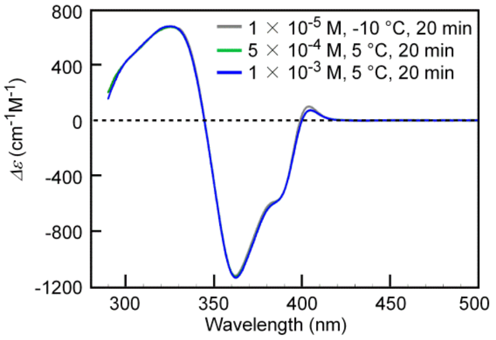

Figure S4. CD spectra of [(*M*)-D-**4**]-C_{12}-TEG in acetone at different concentrations and temperatures.

(a) (b)

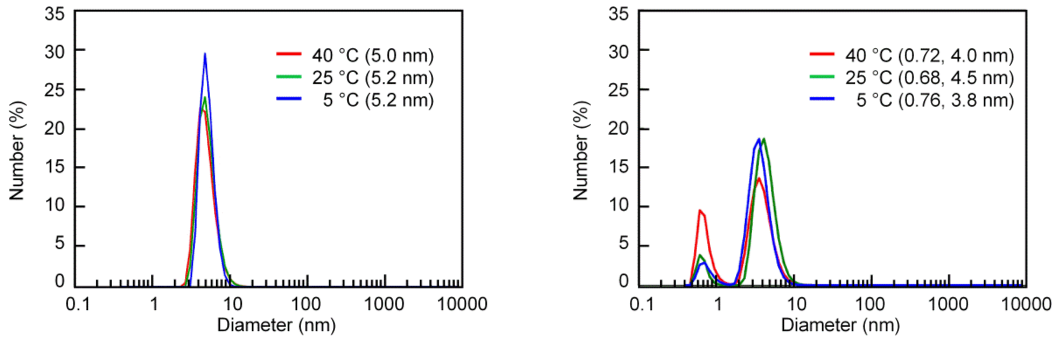

Figure S5. Number average size distributions determined by DLS of [(*M*)-D-**4**]-C_{12}-TEG in acetone at (a) 1×10^{-3} M and (b) 1×10^{-4} M at different temperatures.

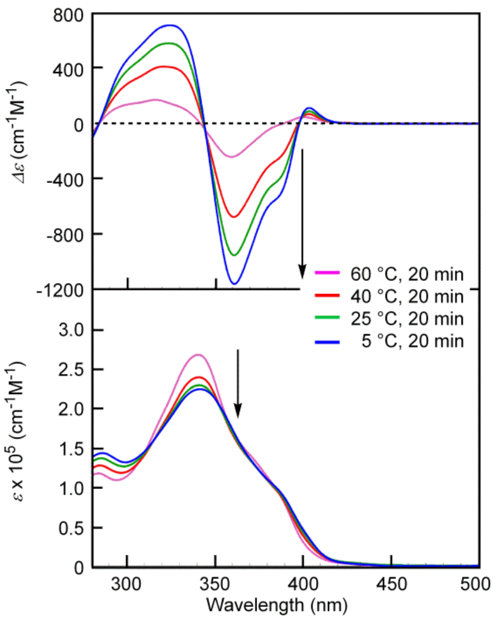

Figure S6. CD (top) and UV-Vis (bottom) spectra of [(*M*)-D-**4**]-C_{12}-TEG in ethyl acetate (5×10^{-4} M) at different temperatures. Arrows show the changes upon cooling.

< CD, UV-Vis, and DLS studies of [(*M*)-D-4]-C_{12}-TEG in acetone/water/triethylamine >

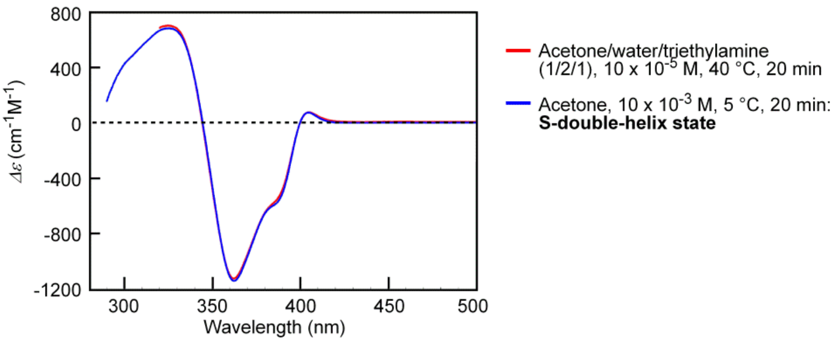

Figure S7. CD spectra of [(*M*)-D-**4**]-C_{12}-TEG in acetone/water/triethylamine (1/2/1, 1 × 10^{-5} M, 40 °C) and acetone (1 × 10^{-3} M, 5 °C).

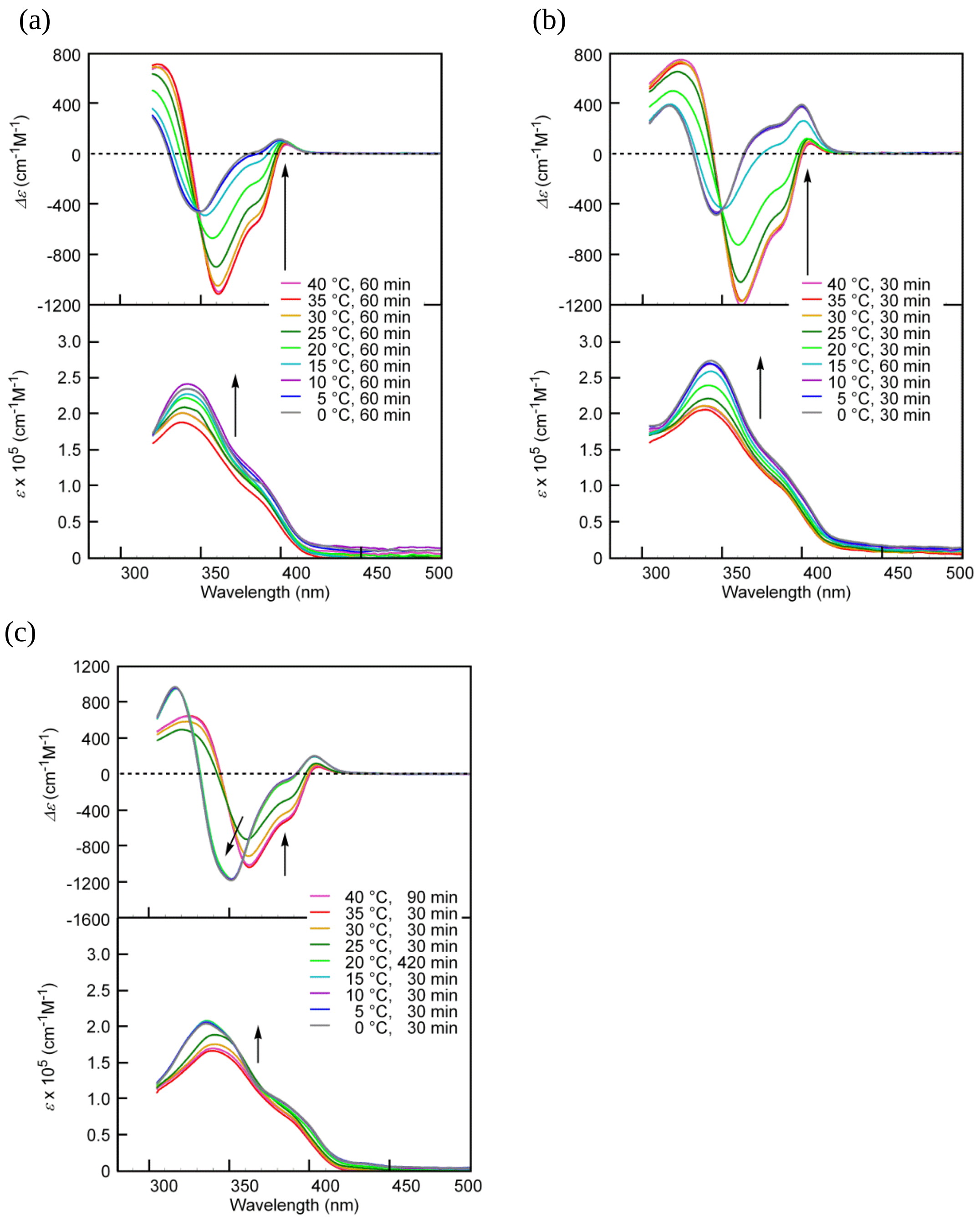

Figure S8. CD (top) and UV-Vis (bottom) spectra of [(*M*)-D-**4**]-C_{12}-TEG in acetone/water/triethylamine (1/2/1) at (a) 5 × 10^{-6} M, (b) 1.5 × 10^{-5} M, and (c) 3 × 10^{-5} M at different temperatures. Arrows show the changes upon cooling. Formation of polymolecular aggregates at 3 × 10^{-5} M below 20 °C was indicated by DLS analysis (Figure S9b).

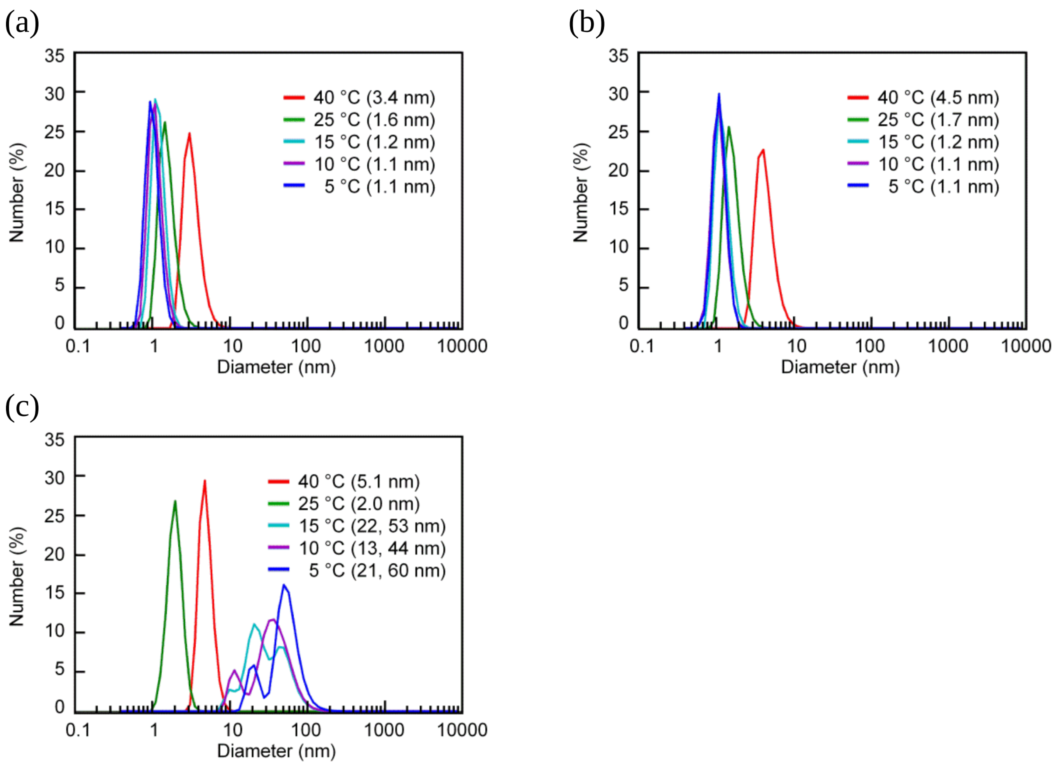

Figure S9. Number average size distributions determined by DLS of [(*M*)-D-**4**]-C_{12}-TEG in acetone/water/triethylamine (1/2/1) at (a) 5×10^{-6} M, (b) 1.5×10^{-5} M, and (c) 3×10^{-5} M at different temperatures.

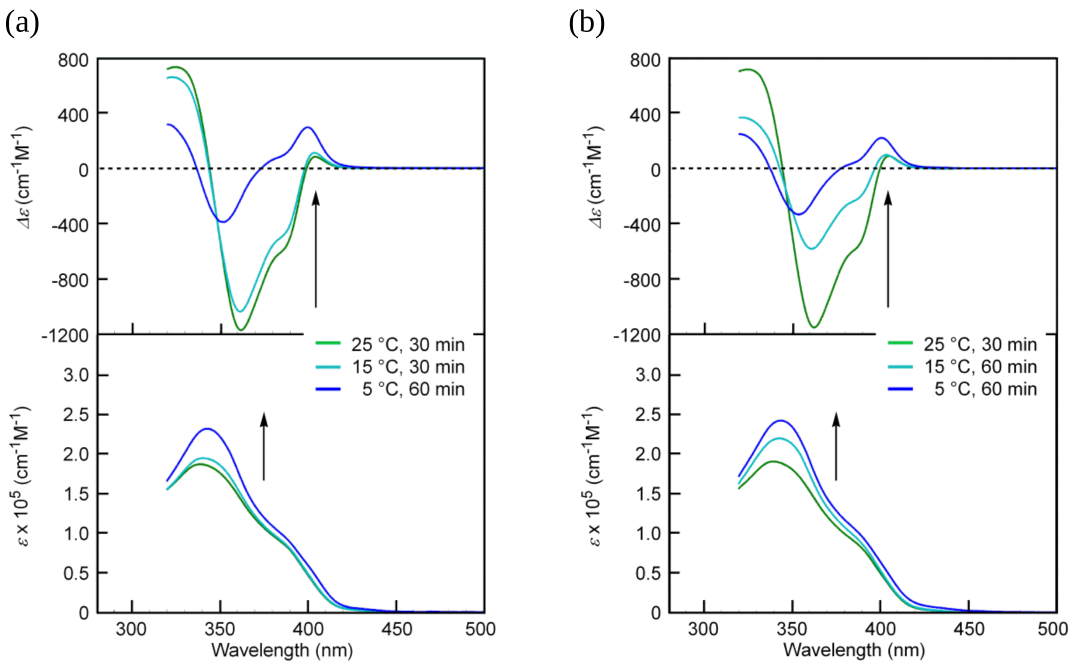

Figure S10. CD (top) and UV-Vis (bottom) spectra of [(*M*)-D-**4**]-C_{12}-TEG in acetone/water/triethylamine (1×10^{-5} M) with the solvent ratio (a) 0.6/2/1 and (b) 0.8/2/1 at different temperatures. Separation of the solvent into triethylamine and water phases occurred in both (a) and (b) at 40 °C. Arrows show the changes upon cooling.

< CD, UV-Vis, and DLS studies of [(*M*)-D-4]-C_{12}-TEG in acetone/triethylamine >

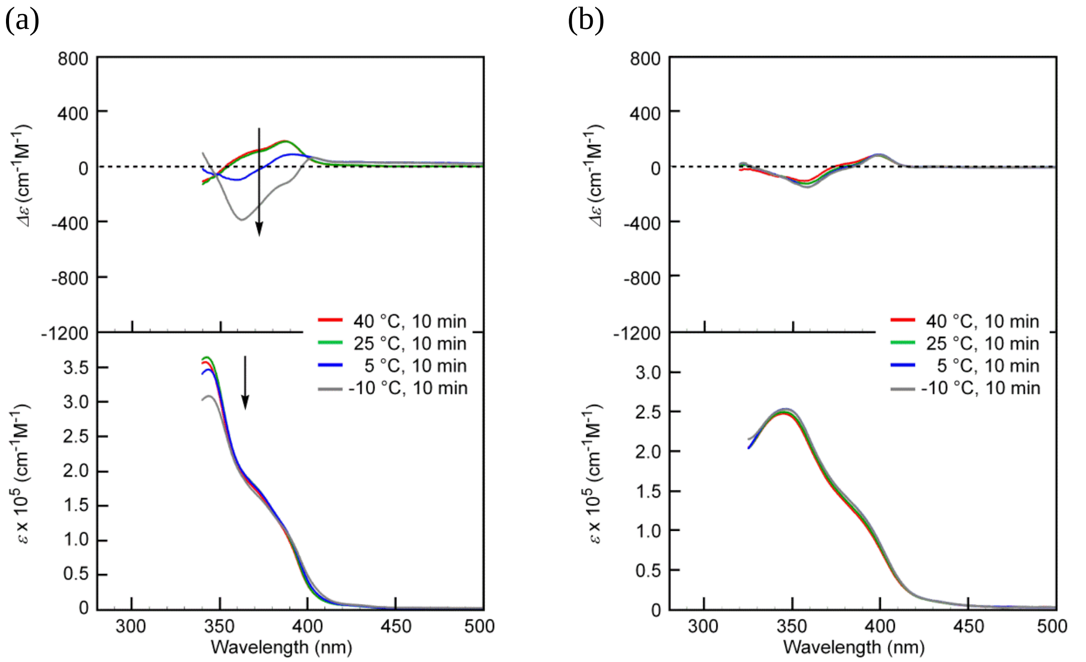

Figure S11. CD (top) and UV-Vis (bottom) spectra of [(*M*)-D-**4**]-C_{12}-TEG in (a) acetone/triethylamine (3/1, 1 × 10^{-5} M) and (b) acetone/water (3/1, 1 × 10^{-5} M)[11] at different temperatures.

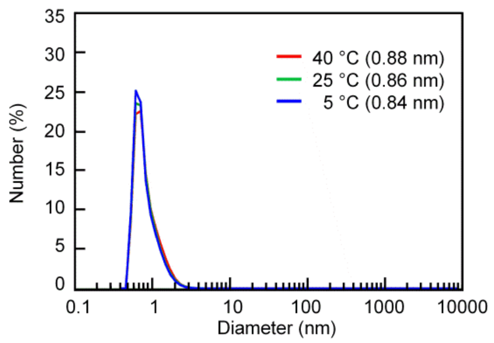

Figure S12. Number average size distributions determined by DLS of [(*M*)-D-**4**]-C_{12}-TEG in acetone/triethylamine (3/1, 1 × 10^{-5} M) at 25 °C. The viscosities and refractive indexes of acetone at each temperature (Table S1) were used.

<Variable-temperature ^{1}H-NMR study of [(*M*)-D-4]-C_{12}-TEG in acetone/water/triethylamine>

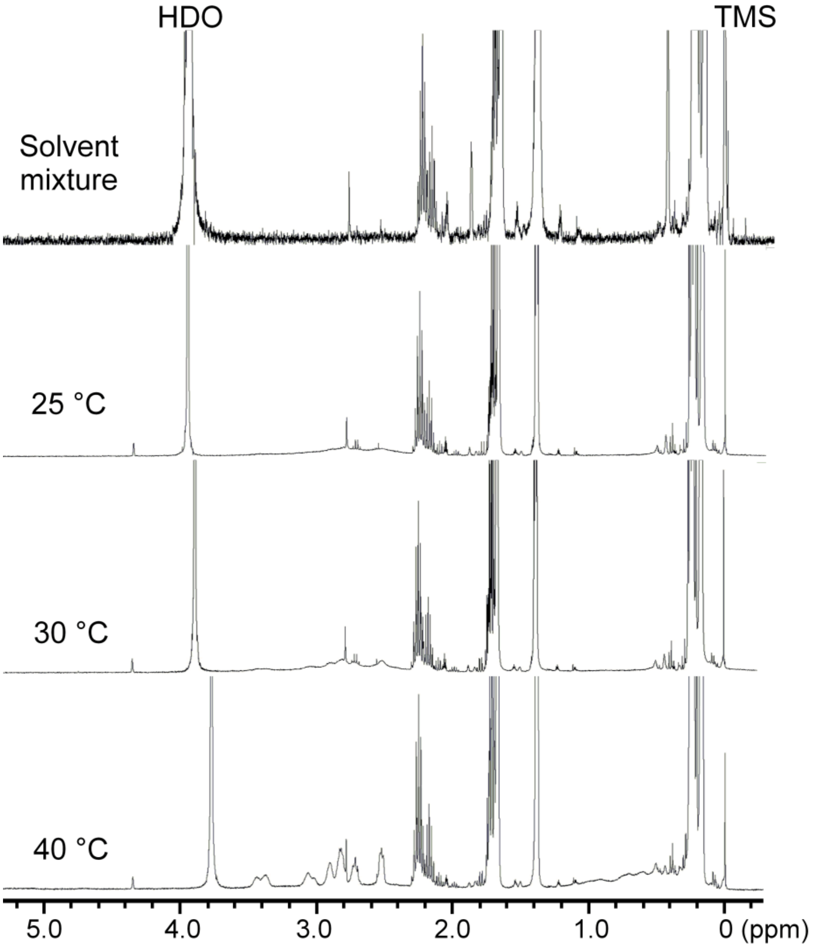

Figure S13. ^{1}H NMR spectra of [(*M*)-D-**4**]-C_{12}-TEG in $CD_3COCD_3/D_2O/N(CD_2CD_3)_3$ (1/2/1, 2 × 10^{-4} M) at different temperatures. The spectra are normalized based on the height of TMS signals.

< Double helix/random coil ratio >

The ratio of the molecules at double helix and random coil state was obtained as follows.

Observed $\Delta\varepsilon_{obs}$ is described in eq. S1 using the value $\Delta\varepsilon_{H}$ of S-double-helix state and $\Delta\varepsilon_{R}$ of S-random-coil state, where x% and y% are the ratio of double helix and random coil, respectively.

$$\Delta\varepsilon_{obs} = \Delta\varepsilon_{H}\,(x/100) + \Delta\varepsilon_{R}\,(y/100) \qquad \text{(eq. S1)}$$

The relationship between x and y is defined as shown in eq. S2.

$$x + y = 100 \qquad \text{(eq. S2)}$$

The equations eq. S3 and eq. S4 are derived from eq. S1 and eq. S2.

$$x = 100\cdot(\Delta\varepsilon_{obs} - \Delta\varepsilon_{R}) / (\Delta\varepsilon_{H} - \Delta\varepsilon_{R}) \qquad \text{(eq. S3)}$$

$$y = 100\cdot(\Delta\varepsilon_{H} - \Delta\varepsilon_{obs}) / (\Delta\varepsilon_{H} - \Delta\varepsilon_{R}) \qquad \text{(eq. S4)}$$

In this study, the $\Delta\varepsilon$ at 360 nm were used. The experimental values $\Delta\varepsilon_{H} = -1.1 \times 10^{3}$ $cm^{-1}M^{-1}$ and $\Delta\varepsilon_{R} = -3.1 \times 10^{2}$ $cm^{-1}M^{-1}$ were obtained from the CD spectra of S-double-helix in acetone (1×10^{-3} M, 5 °C, 20 min: Figure 2) and S_{aq}-random-coil state in acetone/water/triethylamine (1/2/1, 1×10^{-5} M, 5 °C, 60 min: Figure 3a), respectively. The CD spectrum of S_{aq}-random-coil state was slightly different from that of random-coil state in organic solvents in intensity and the wavelength (Figure S1, 40 °C), which may be due to a restricted conformation of random coils in the aqueous medium by hydration at the TEG moieties.

Using $\Delta\varepsilon_{obs}$ observed in acetone/water/triethylamine (1/2/1, 1×10^{-5} M) at each temperature, yields x and y were determined (Figure 3a and Table S4).

< Thermodynamic parameters of double-helix formation of [(*M*)-D-4]-C_{12}-TEG in acetone/water/triethylamine >

The double-helix formation of [(*M*)-D-**4**]-C_{12}-TEG (1×10^{-5} M) in acetone/water/triethylamine (1/2/1) is shown with the following equation (eq. S5), where R and H are random coil and double helix, respectively.

$$\mathrm{R} + \mathrm{R} \rightleftarrows \mathrm{H} \qquad \text{(eq. S5)}$$

Dimerization constant *K*, enthalpy change Δ*H*, and entropy change Δ*S* were obtained using the CD $\Delta\varepsilon$ values as below, following the reported method.[S3]

Dimerization constant *K* is defined as shown in eq. S6, where [R] and [H] are random coil and double helix concentration at equilibrium, respectively.

$$K = [\mathrm{H}]/ [\mathrm{R}]^2 \qquad \text{(eq. S6)}$$

Observed $\Delta\varepsilon_{obs}$ is described in eq. S7 using the concentration of [(*M*)-D-**4**]-C_{12}-TEG [C], the $\Delta\varepsilon$ value of S-double-helix state $\Delta\varepsilon_{H}$, and the $\Delta\varepsilon$ value of S-random-coil state $\Delta\varepsilon_{R}$.

$$\Delta\varepsilon_{obs} = \Delta\varepsilon_{H} (2[\mathrm{H}]/ [\mathrm{C}]) + \Delta\varepsilon_{R}([\mathrm{R}]/[\mathrm{C}]) \qquad \text{(eq. S7)}$$

The concentration [C] is defined as shown in eq S8.

$$[\mathrm{C}] = 2[\mathrm{H}] + [\mathrm{R}] \qquad \text{(eq. S8)}$$

The rearrangement of eq. S8 gives eq. S9.

$$[\mathrm{R}] = [\mathrm{C}] - 2[\mathrm{H}] \qquad \text{(eq. S9)}$$

Substituting [R] in eq. S7 with eq. S9, and solving for [H] gives eq. S10

$$[\mathrm{H}] =(1/2) \cdot [\mathrm{C}] (\Delta\varepsilon_{obs} - \Delta\varepsilon_{R})/(\Delta\varepsilon_{H} - \Delta\varepsilon_{R}) \qquad \text{(eq. S10)}$$

In this study, the $\Delta\varepsilon$ at 360 nm were used to obtain Δ*H* and Δ*S*. The values $\Delta\varepsilon_{H} = -1.1 \times 10^3$ $\mathrm{cm^{-1}M^{-1}}$ and $\Delta\varepsilon_{R} = -3.1 \times 10^2$ $\mathrm{cm^{-1}M^{-1}}$ were obtained from the CD spectra of S-double-helix in acetone (1×10^{-3} M, 5 °C, 20 min: Figure 2) and S_{aq}-random-coil state in acetone/water/triethylamine (1/2/1, 1×10^{-5} M, 5 °C, 60 min: Figure 3a), respectively. [C] = 1×10^{-5} M. *K* was calculated according to eq. S6, S8, and S10 using $\Delta\varepsilon_{obs}$ in acetone/water/triethylamine (1/2/1, 1×10^{-5} M) at different temperatures (Table S4).

The van't Hoff plots using the *K* values (1.7×10^6 $\mathrm{M^{-1}}$ at 30 °C, 6.4×10^5 $\mathrm{M^{-1}}$ at 27.5 °C, 2.6×10^5 $\mathrm{M^{-1}}$ at 25 °C, 1.1×10^5 $\mathrm{M^{-1}}$ at 22.5 °C, 4.3×10^4 $\mathrm{M^{-1}}$ at 20 °C, 2.1×10^4 $\mathrm{M^{-1}}$ at 17.5 °C, and 1.2×10^4 $\mathrm{M^{-1}}$ at 15 °C) provided $\Delta H = +2.4 \times 10^2$ $\mathrm{kJ \cdot mol^{-1}}$ and $\Delta S = +9.2 \times 10^2$ $\mathrm{J \cdot mol^{-1} \cdot K^{-1}}$ (Figure S13).

Table S4. The double helix/random coil ratio and equilibrium constant K of [(M)-D-**4**]-C_{12}-TEG in acetone/water/triethylamine (1/2/1, 1 × 10^{-5} M) at different temperatures.

Temp. (°C)	$\Delta\varepsilon_{obs}$ at 360 nm ($cm^{-1}M^{-1}$)	Double helix	Random coil	K (M^{-1})
40	-1.0×10^3	90%	10%	4.3×10^6
35	-1.0×10^3	92%	8%	7.0×10^6
30	-9.8×10^2	84%	16%	1.7×10^6
27.5	-9.1×10^2	76%	24%	6.4×10^5
25	-8.2×10^2	65%	35%	2.6×10^5
22.5	-7.1×10^2	51%	49%	1.1×10^5
20	-5.9×10^2	36%	64%	4.3×10^4
17.5	-5.0×10^2	24%	76%	2.1×10^4
15	-4.4×10^2	17%	83%	1.2×10^4
12.5	-3.7×10^2	8%	92%	4.6×10^3
10	-3.3×10^2	3%	97%	1.3×10^3
5	-3.1×10^2	< 1%	> 99%	—

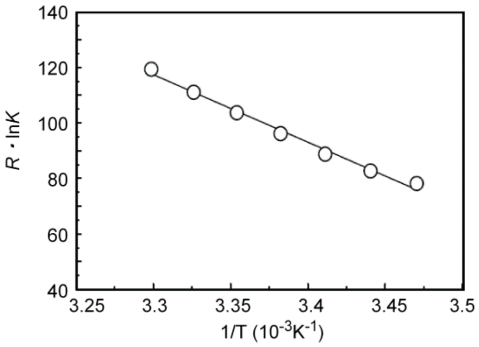

$$R \cdot \ln K = -\Delta H/T + \Delta S$$

ΔH = +2.4 x 10^2 kJ · mol^{-1}
ΔS = +9.2 x 10^2 J · $mol^{-1}K^{-1}$
(R^2 = 0.99)

Figure S14. The van’t Hoff plots for the double-helix formation of [(M)-D-**4**]-C_{12}-TEG in acetone/water/triethylamine (1/2/1, 1 × 10^{-5} M).

3,4,5-Tris{2-[2-(2-methoxyethoxy)ethoxy]ethoxy} benzoic acid 12-{[(4-methylphenyl)sulfonyl] oxy}dodecyl ester 3. ^{1}H NMR ($CDCl_3$, r.t., 400 MHz)

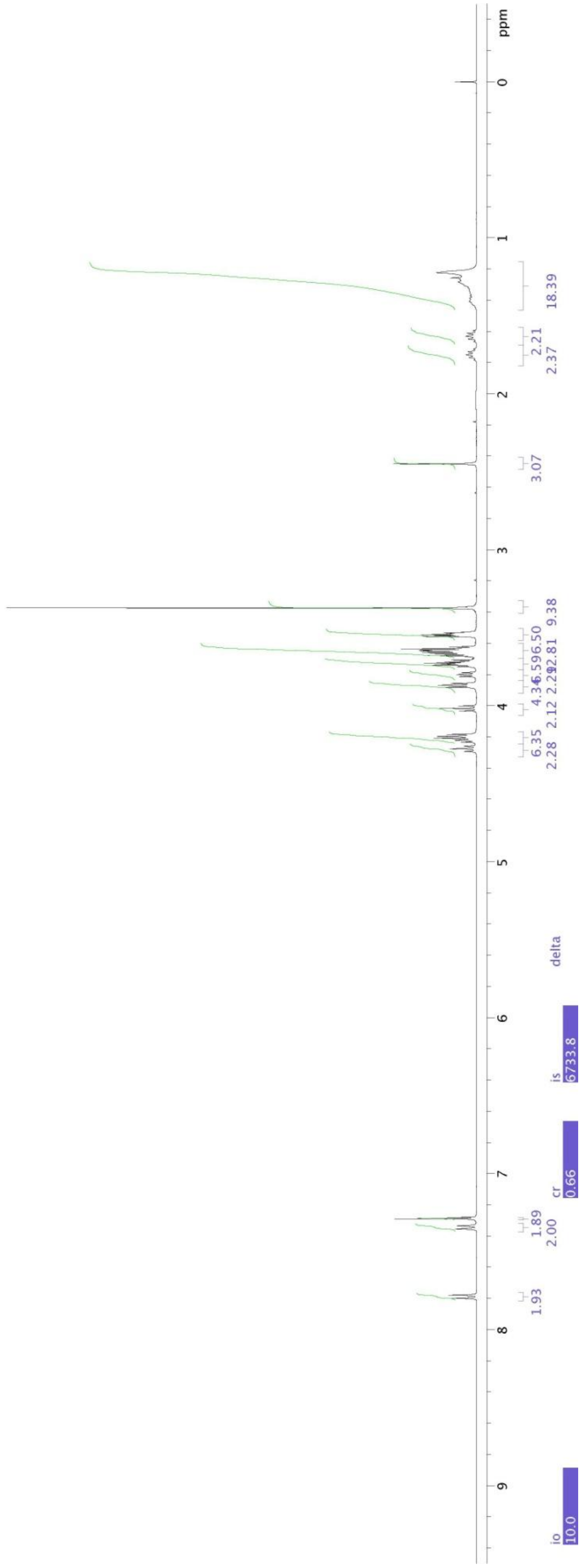

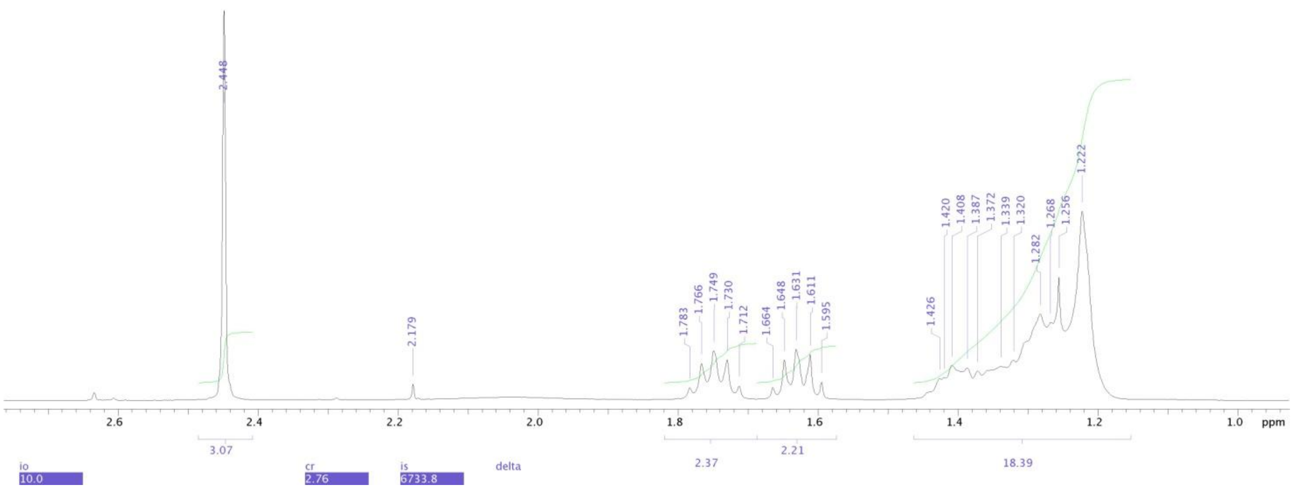

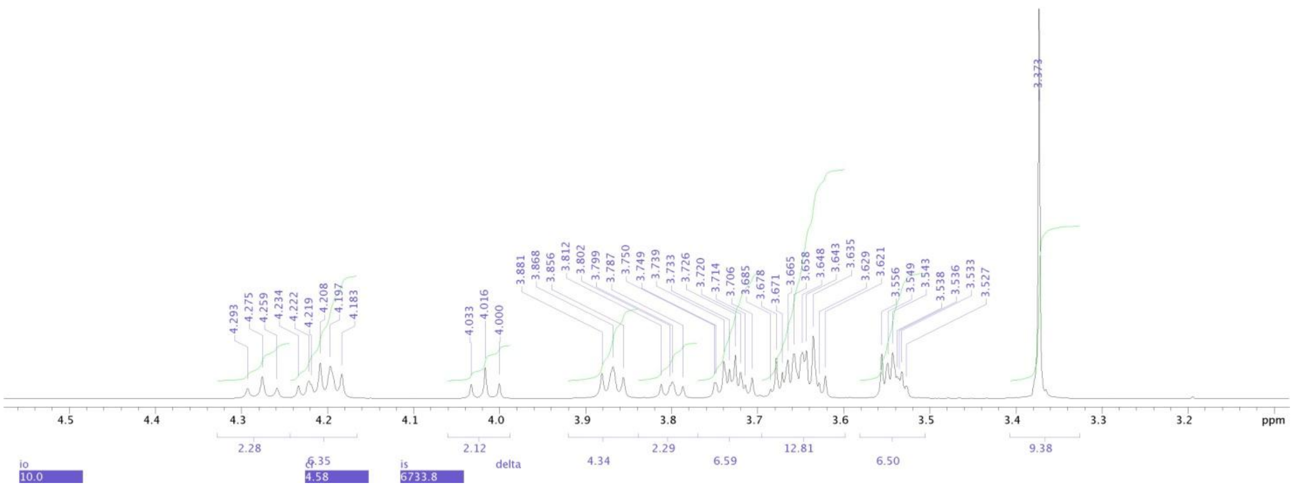

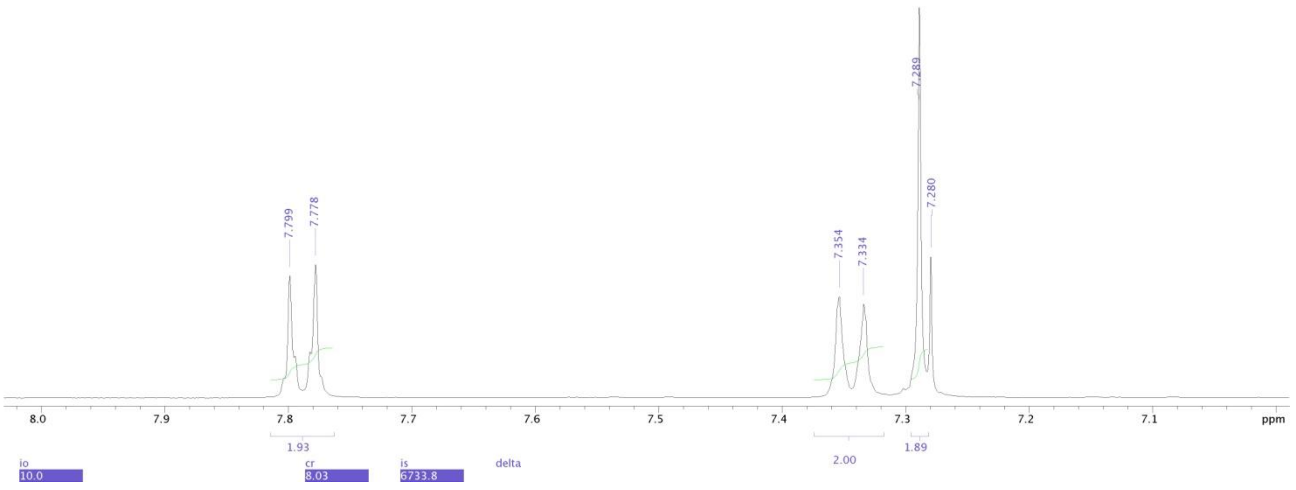

3,4,5-Tris{2-[2-(2-methoxyethoxy)ethoxy]ethoxy} benzoic acid 12-{[(4-methylphenyl)sulfonyl] oxy}dodecyl ester 3. ^{13}C NMR ($CDCl_3$, r.t., 100 MHz)

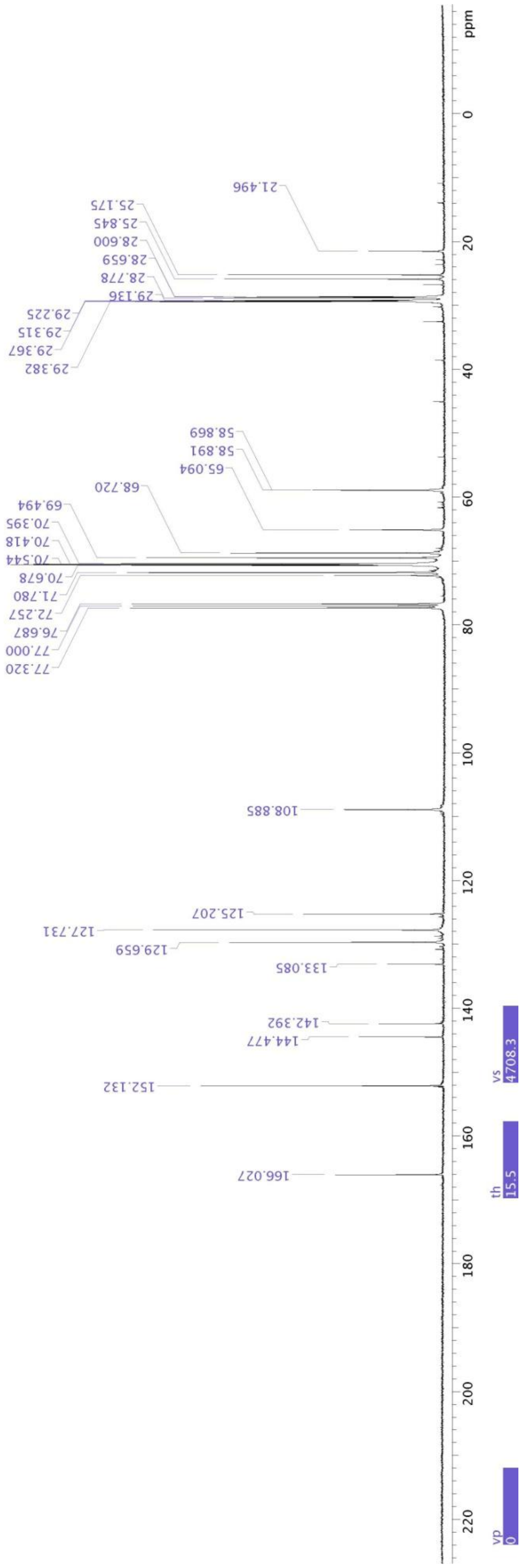

3,4,5-Tris{2-[2-(2-methoxyethoxy)ethoxy]ethoxy} benzoic acid 12-(4-iodophenoxy)dodecyl ester 4. ^{1}H NMR ($CDCl_3$, r.t., 400 MHz)

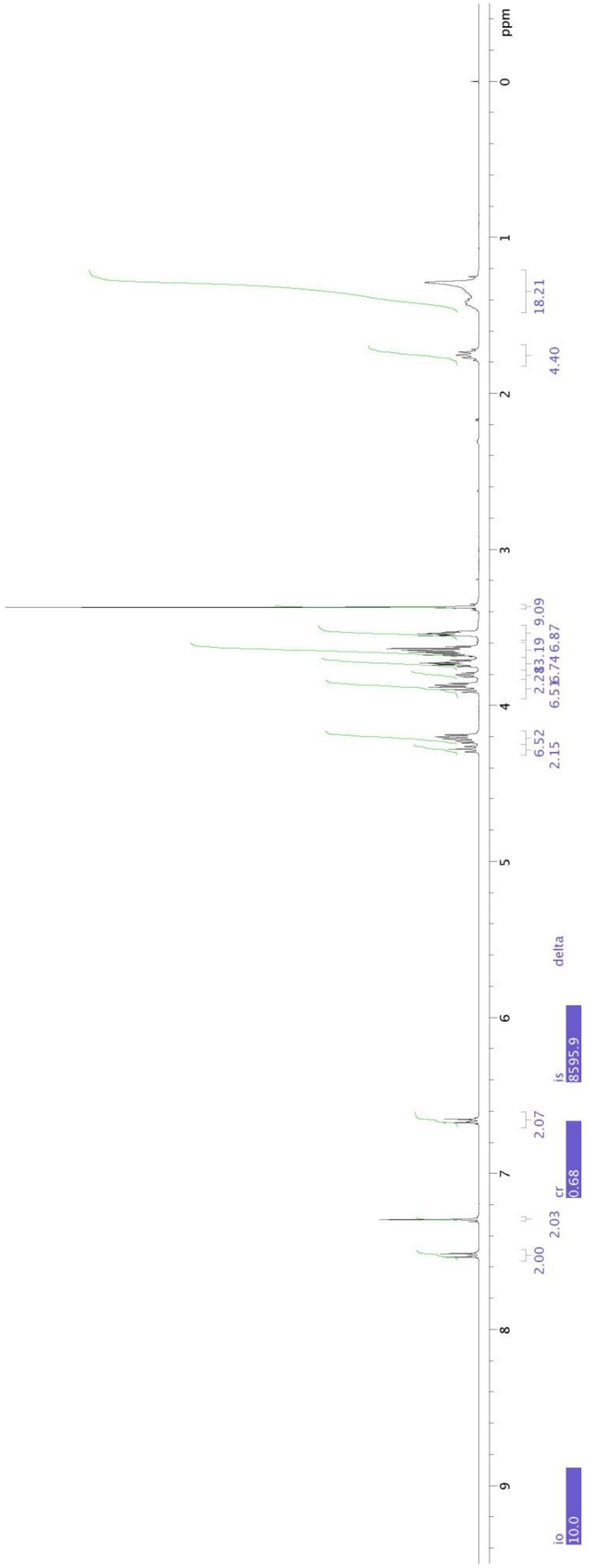

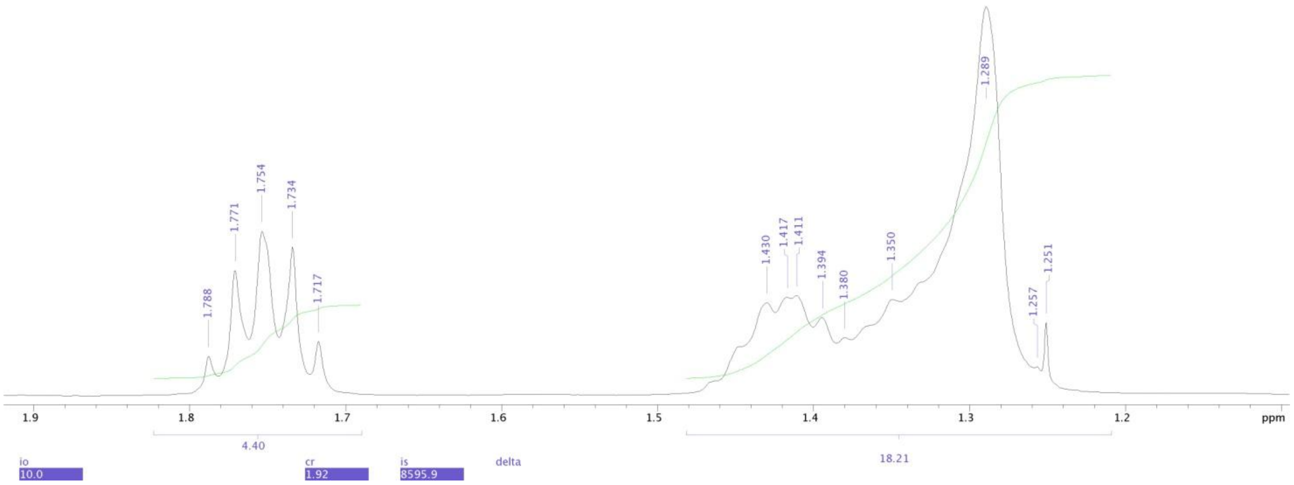

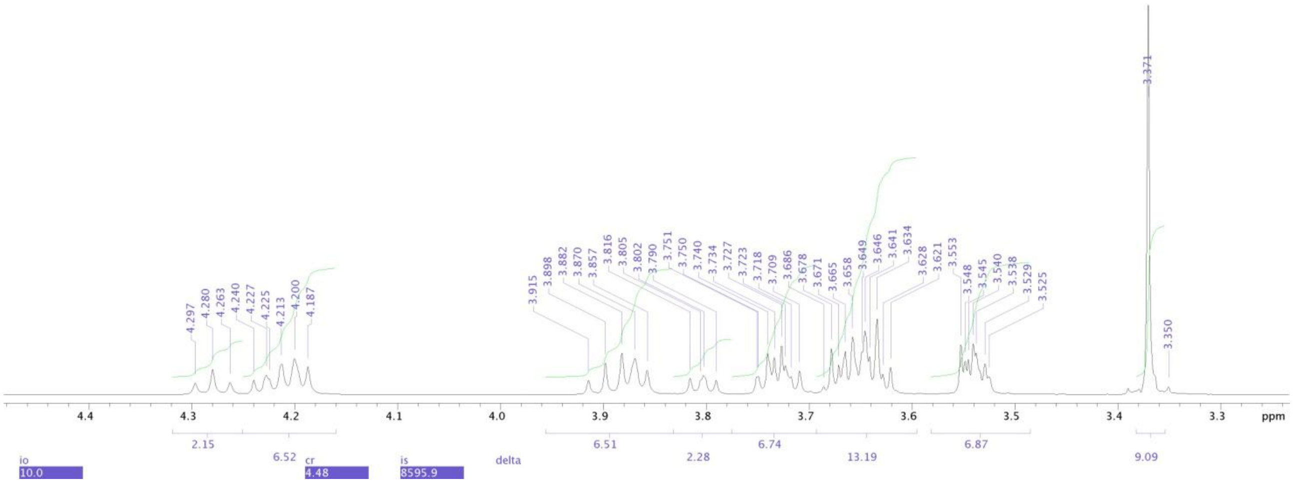

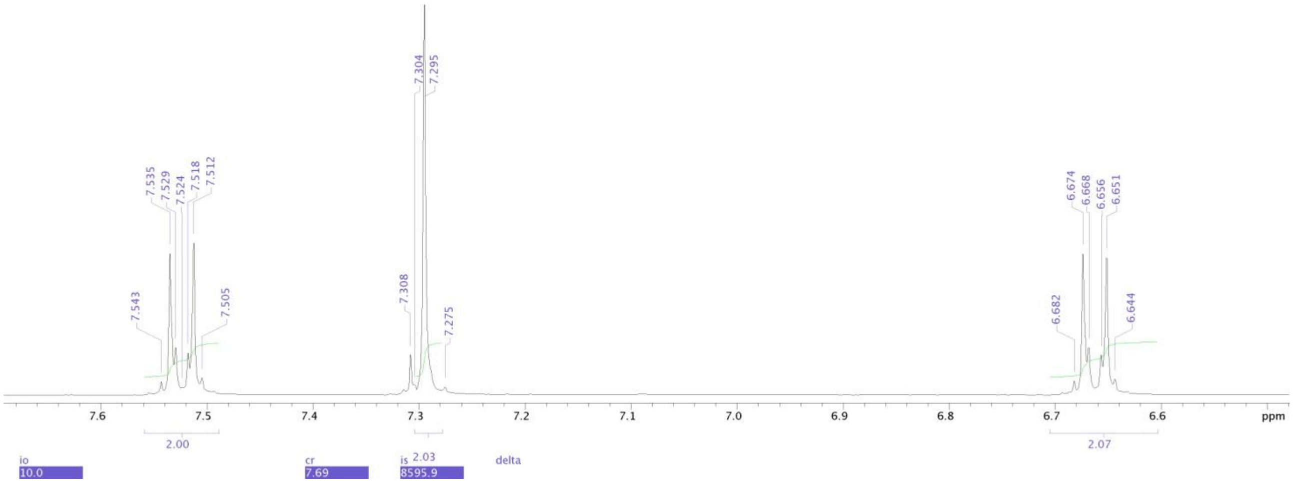

3,4,5-Tris{2-[2-(2-methoxyethoxy)ethoxy]ethoxy} benzoic acid 12-(4-iodophenoxy)dodecyl ester 4. ^{13}C NMR ($CDCl_3$, r.t., 100 MHz)

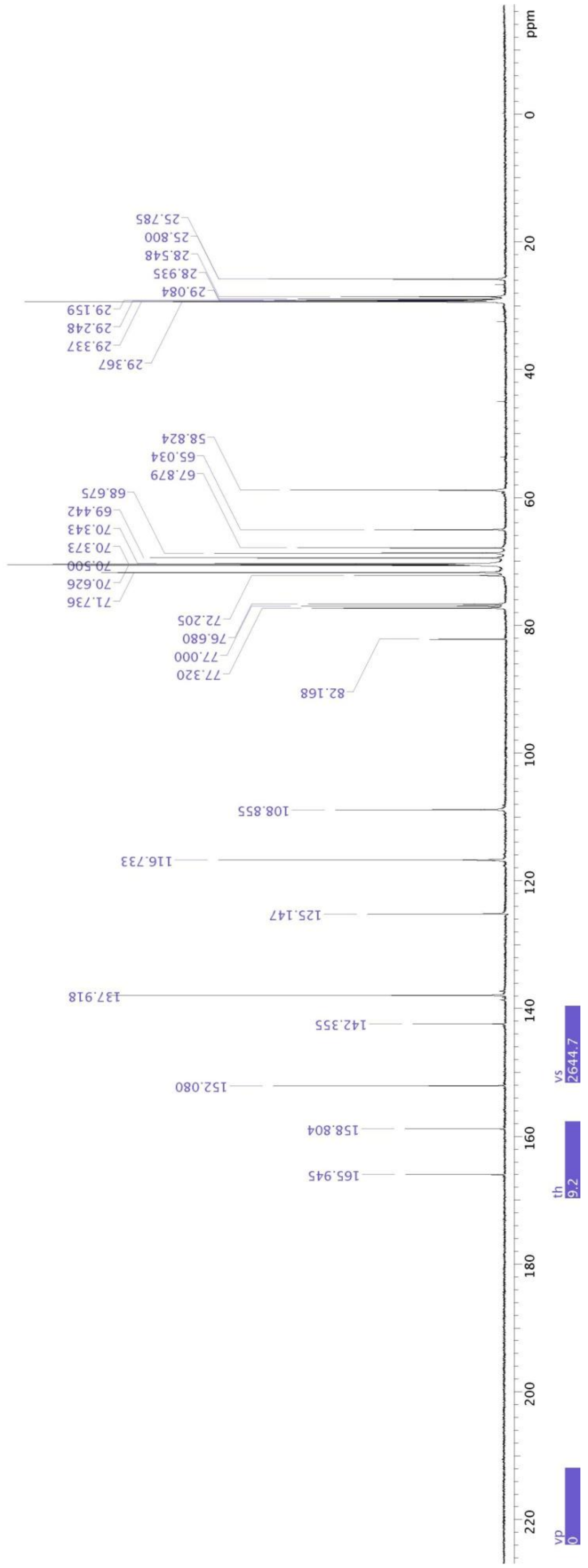

(*M*)-Ethynylhelicene tetramer with PEG terminal groups, [(*M*)-D-4]-C_{12}-TEG. ^{1}H NMR ($CDCl_3$, r.t., 400 MHz)

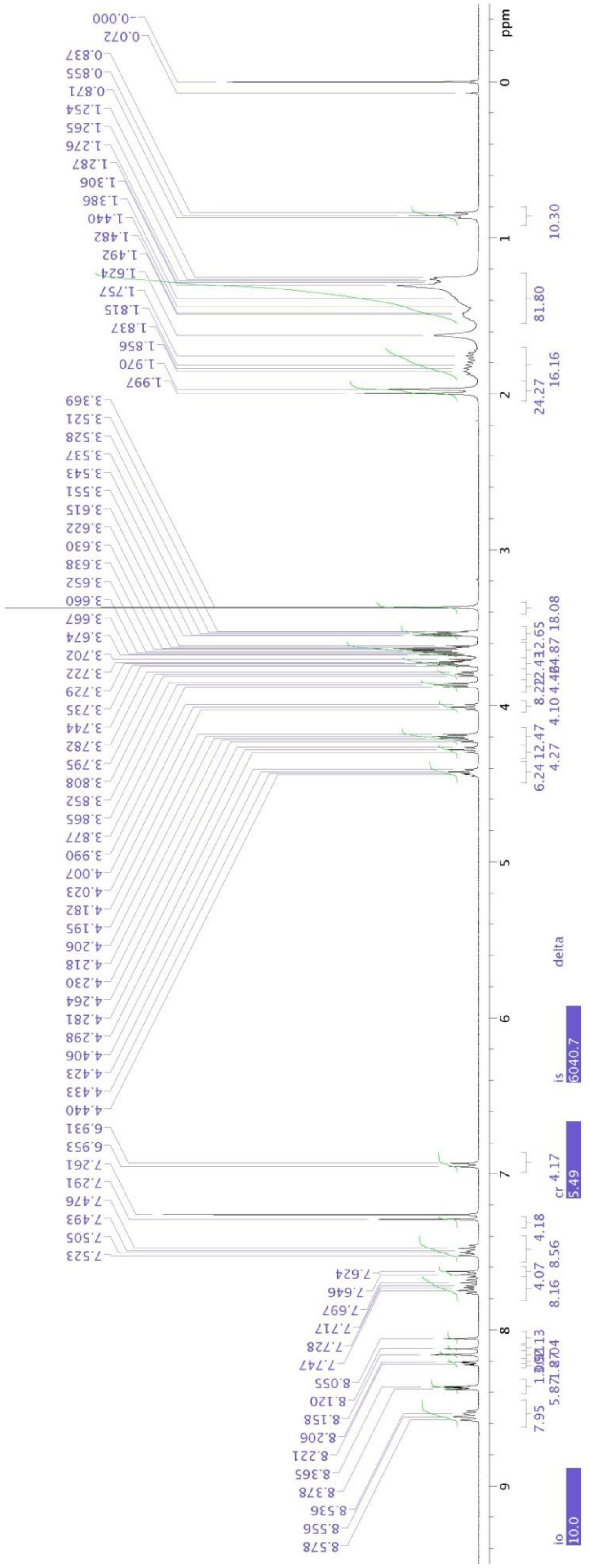

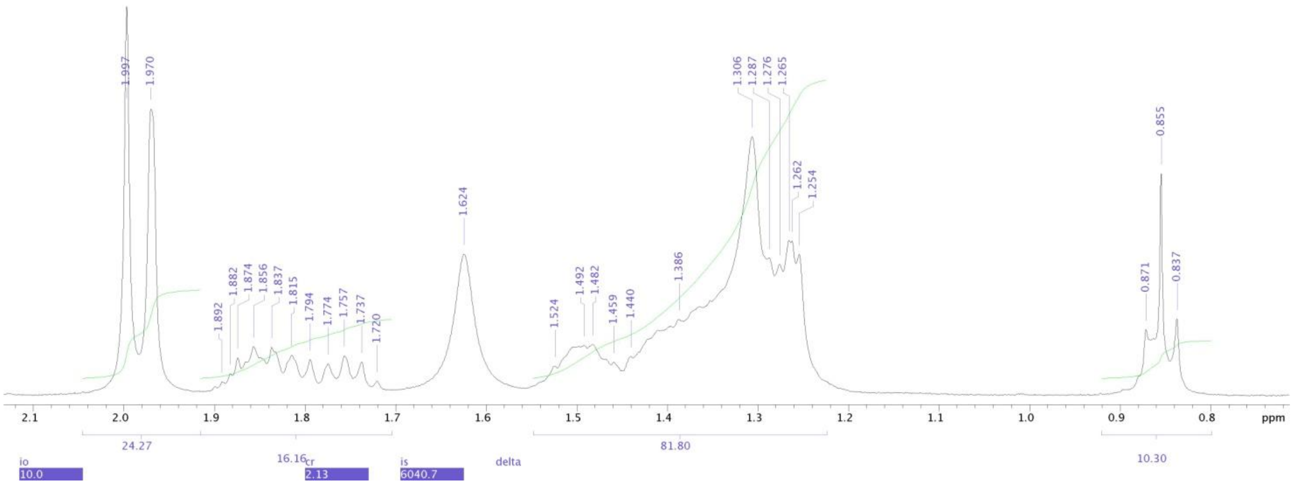
1.997
1.970
1.892
1.882
1.874
1.856
1.837
1.815
1.794
1.774
1.757
1.737
1.720
1.624
1.524
1.492
1.482
1.459
1.440
1.386
1.306
1.287
1.276
1.265
1.262
1.254
0.871
0.855
0.837
2.1 2.0 1.9 1.8 1.7 1.6 1.5 1.4 1.3 1.2 1.1 1.0 0.9 0.8 ppm
24.27
16.16
81.80
10.30
io 10.0
cr 2.13
is 6040.7
delta

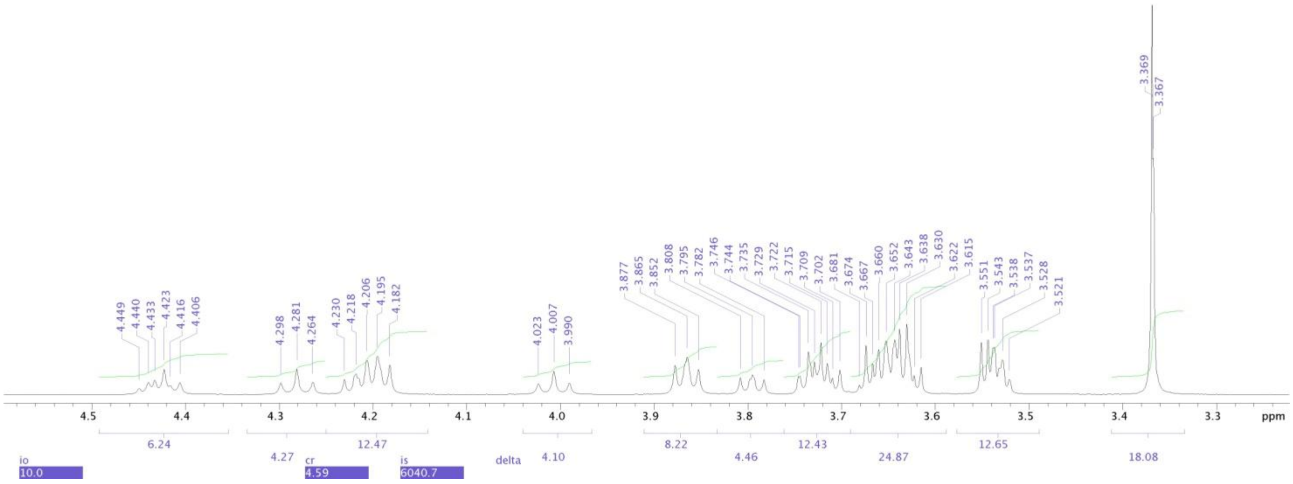
4.449
4.440
4.433
4.423
4.416
4.406
4.298
4.281
4.264
4.230
4.218
4.206
4.195
4.182
4.023
4.007
3.990
3.877
3.865
3.852
3.808
3.795
3.782
3.746
3.744
3.735
3.729
3.722
3.715
3.709
3.702
3.681
3.674
3.667
3.660
3.652
3.643
3.638
3.630
3.622
3.615
3.551
3.543
3.538
3.537
3.528
3.521
3.369
3.367
4.5 4.4 4.3 4.2 4.1 4.0 3.9 3.8 3.7 3.6 3.5 3.4 3.3 ppm
6.24
4.27
12.47
4.10
8.22
4.46
12.43
24.87
12.65
18.08
io 10.0
cr 4.59
is 6040.7
delta

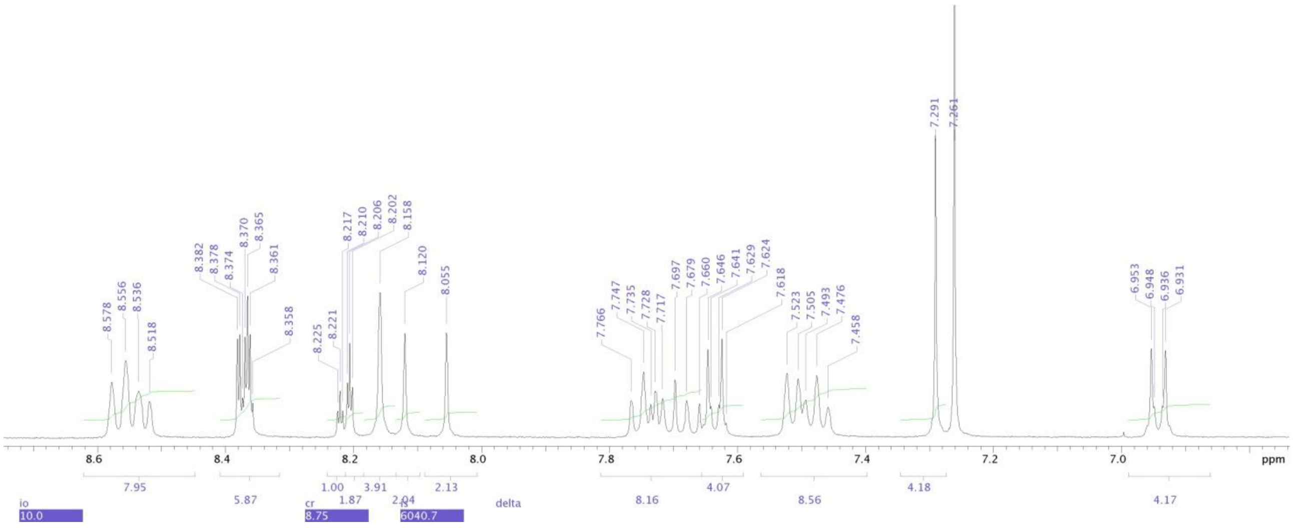
8.578
8.556
8.536
8.518
8.382
8.378
8.374
8.370
8.365
8.361
8.358
8.225
8.221
8.217
8.210
8.206
8.202
8.158
8.120
8.055
7.766
7.747
7.735
7.728
7.717
7.697
7.679
7.660
7.646
7.641
7.629
7.624
7.618
7.523
7.505
7.493
7.476
7.458
7.291
7.261
6.953
6.948
6.936
6.931
8.6 8.4 8.2 8.0 7.8 7.6 7.4 7.2 7.0 ppm
7.95
5.87
1.00
1.87
3.91
2.04
2.13
8.16
4.07
8.56
4.18
4.17
io 10.0
cr 8.75
is 6040.7
delta

(*M*)-Ethynylhelicene tetramer with PEG terminal groups, [(*M*)-D-4]-C_{12}-TEG. ^{13}C NMR ($CDCl_3$, r.t., 100 MHz)

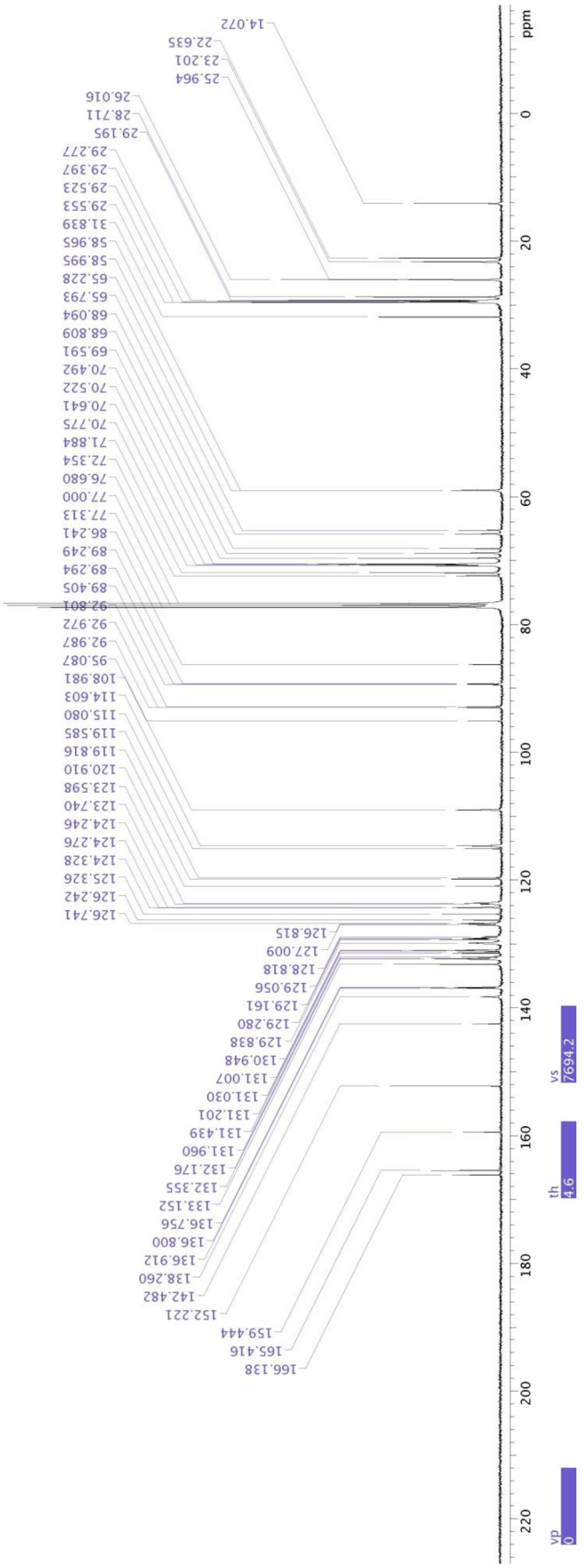

< References >

S1) Münzenberg, C.; Rossi, A.; Feldman, K.; Fiolka, R.; Stermmer, A.; Kita-Tokarczyk, K.; Meier, W.; Sakamoto, J.; Lukin, O.; Schülter, A. D. *Chem. Eur. J.* **2008**, *14*, 10797−10807.

S2) Sugiura, H.; Nigorikawa, Y.; Saiki, Y.; Nakamura, K.; Yamaguchi, M. *J. Am. Chem. Soc.* **2004**, *126*, 14858−14864.

S3) Sugiura, H.; Amemiya, R.; Yamaguchi, M. *Chem. Asian J.* **2008**, *3*, 244−260.
